# Application of physiologically based pharmacokinetic modeling to understand real‐world outcomes in patients receiving imatinib for chronic myeloid leukemia

**DOI:** 10.1002/prp2.1082

**Published:** 2023-07-07

**Authors:** Josephine A. Adattini, Jeffry Adiwidjaja, Annette S. Gross, Andrew J. McLachlan

**Affiliations:** ^1^ Sydney Pharmacy School, Faculty of Medicine and Health The University of Sydney Sydney Australia; ^2^ Clinical Pharmacology Modelling & Simulation, GlaxoSmithKline R&D Sydney Australia

**Keywords:** adverse drug reactions, drug interactions, early molecular response, exposure‐response, imatinib, physiologically based pharmacokinetic (PBPK), simulation

## Abstract

We aimed to use physiologically based pharmacokinetic (PBPK) modeling and simulation to predict imatinib steady‐state plasma exposure in patients with chronic myeloid leukemia (CML) to investigate variability in outcomes. A validated imatinib PBPK model (Simcyp Simulator) was used to predict imatinib AUC_ss_, C_ss,min_ and C_ss,max_ for patients with CML (*n* = 68) from a real‐world retrospective observational study. Differences in imatinib exposure were evaluated based on clinical outcomes, (a) Early Molecular Response (EMR) achievement and (b) occurrence of grade ≥3 adverse drug reactions (ADRs), using the Kruskal‐Wallis rank sum test. Sensitivity analyses explored the influence of patient characteristics and drug interactions on imatinib exposure. Simulated imatinib exposure was significantly higher in patients who achieved EMR compared to patients who did not (geometric mean AUC_0‐24,ss_ 51.2 vs. 42.7 μg h mL^−1^, *p* < 0.05; C_ss,min_ 1.1 vs. 0.9 μg mL^−1^, *p* < 0.05; C_ss,max_ 3.4 vs. 2.8 μg mL^−1^, *p* < 0.05). Patients who experienced grade ≥3 ADRs had a significantly higher simulated imatinib exposure compared to patients who did not (AUC_0‐24,ss_ 56.1 vs. 45.9 μg h mL^−1^, *p* < 0.05; C_ss,min_ 1.2 vs. 1.0 μg mL^−1^, *p* < 0.05; C_ss,max_ 3.7 vs. 3.0 μg mL^−1^, *p* < 0.05). Simulations identified a range of patient (sex, age, weight, abundance of hepatic CYP2C8 and CYP3A4, α_1_‐acid glycoprotein concentrations, liver and kidney function) and medication‐related factors (dose, concomitant CYP2C8 modulators) contributing to the inter‐individual variability in imatinib exposure. Relationships between imatinib plasma exposure, EMR achievement and ADRs support the rationale for therapeutic drug monitoring to guide imatinib dosing to achieve optimal outcomes in CML.

## Graphical abstract

Combining real word clinical data and pharmacokinetic modeling, this study investigated the exposure‐response relationship for imatinib in people with chronic myeloid leukemia. Patients with higher imatinib exposure were more likely to achieve clinical response, but also the risk of imatinib‐related harms.

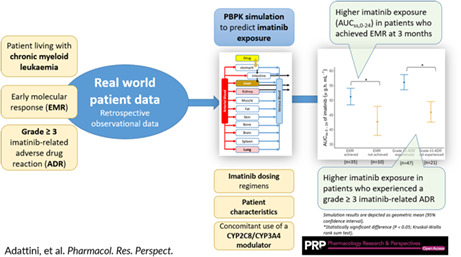

**Abbreviations**
AAG: α_1_‐acid glycoprotein
ADR: adverse‐drug reaction
ADRs: adverse drug reactions
AUC_ss_
: area under the concentration‐time curve at steady state
BSA: body surface area
CI: confidence interval
CL_R_
: renal clearance
CML: chronic myeloid leukemia
DMR: deep molecular response
EMR: Early Molecular Response
fu_p_
: unbound fraction in plasma
HLM: human liver microsomes
MMR: major molecular response
OR: Odds ratio
PBPK: physiologically based pharmacokinetic
PK: pharmacokinetics
SD: standard deviation
sDMR: sustained deep molecular response
TDM: therapeutic drug monitoring
TKI: tyrosine kinase inhibitor

## 1 INTRODUCTION

Imatinib, a *BCR‐* *ABL* tyrosine kinase inhibitor (TKI), has significantly changed the treatment landscape of Philadelphia chromosome‐positive chronic myeloid leukemia (CML). Despite showing significantly superior survival outcomes and improved tolerability compared to interferon‐α plus low‐dose cytarabine,^1^ many patients need to discontinue imatinib treatment and switch to another TKI.^2^ We recently conducted a retrospective observational study of real‐world patients with CML in Australia and observed that 57% (95% confidence interval [CI], 46–66%) of patients had experienced an imatinib‐related adverse‐drug reaction (ADR) necessitating imatinib dose changes or discontinuation, and 53% (95% CI, 42–63%) a grade ≥3 ADR, by 18 months of treatment.^2^ These patients required increased monitoring and utilization of healthcare services, with many needing to commence new long‐term medicines for the management of imatinib‐related ADRs.^2^ A large proportion of patients never achieved sustained deep molecular response (sDMR; *BCR‐ABL1* ^*IS*^ ≤0.01% maintained for at least 2 consecutive years) with imatinib treatment (5‐year cumulative incidence of 41%; 95% CI, 27–55%)^2^; a minimum requirement for attempting TKI treatment discontinuation.^3^, ^4^ Furthermore, one in four patients failed to achieve Early Molecular Response (EMR; *BCR‐ABL1* ^*IS*^ ≤ 10%) by 3 months of imatinib treatment^2^; an endpoint predictive for major molecular response (MMR), deep molecular response (DMR) and sDMR achievement.^2^, ^5^, ^6^

Inter‐individual variability in imatinib pharmacokinetics (PK) is a possible determinant of variability in imatinib response.^7^ Large inter‐individual variability has been observed in imatinib trough plasma concentrations at steady‐state (C_ss,min_) in patients administered the same dose, with reported coefficient of variations (CV%) of 40 to 60%.^8^, ^9^, ^10^, ^11^, ^12^ As a drug with a low hepatic extraction ratio, steady‐state total plasma imatinib concentrations are determined by variability in intrinsic hepatic clearance (metabolism and transport) and plasma protein binding.^13^ Both imatinib and its major metabolite N‐desmethyl imatinib undergo mostly hepatic elimination, with biotransformation of imatinib to N‐desmethyl imatinib by cytochrome P450 (CYP)3A4 and CYP2C8 a clinically important inactivating process.^7^, ^13^ Imatinib is also a substrate of P‐glycoprotein (P‐gp) and breast cancer resistant protein (BCRP) efflux transporters, which contribute to the net intestinal absorption of imatinib (via expression of transporters on apical/luminal membrane of enterocytes), biotransformation and excretion of imatinib (via expression of transporters on the bile caniculi of hepatocytes),^7^, ^13^ and retention of imatinib in target cancer cells.^14^, ^15^, ^16^, ^17^ The expression and activities of drug‐metabolizing enzymes and transporters, which can be affected by genetic or environmental factors (e.g., concomitant medicine use and diet),^18^ are hypothesized to influence imatinib plasma and intracellular concentrations, and thus the efficacy and tolerability of imatinib. Patients receiving imatinib treatment are likely to be receiving multiple concomitant medicines for management of their comorbidities (and treatment related ADRs), due to the older median age at CML diagnosis and expected longer survival of patients treated with TKIs.^19^, ^20^, ^21^ As a basic compound, imatinib exhibits concentration‐dependent binding with high affinity to α_1_‐acid glycoprotein (AAG), with an unbound fraction in plasma (fu_p_) of 0.05.^22^ As an acute phase protein, AAG demonstrates highly variable concentrations, increasing 2 to 6‐fold in response to injury, infection, inflammation and cancer.^22^, ^23^ Furthermore, there are different genetic polymorphisms of AAG, with different binding properties of variants reported.^24^

Mechanistic modeling and simulation approaches can be used for predicting and understanding the PK of a drug. Physiologically based pharmacokinetic (PBPK) modeling integrates drug‐specific parameters (e.g., plasma protein binding and clearance) and drug independent system‐specific parameters (e.g., population variables such as organ blood flow, organ volumes and tissue composition) to simulate the time course of drug concentrations in different clinical populations.^25^, ^26^ Using a PBPK model, the drug concentration‐time profile from a specific dose regimen, in a patient from a population for which clinical data was not available, can be predicted. The concentration‐time profile is predicted by considering key determinants of PK in an individual patient, such as geographic ancestry, weight, age, kidney and hepatic function, and genetic polymorphisms in drug‐metabolizing enzymes or transporters. PBPK modeling can also be used to understand possible drug–drug interactions,^25^, ^26^, ^27^, ^28^, ^29^, ^30^ and to predict characteristics that influence inter‐individual variability in drug exposure.^31^, ^32^ Identifying factors that contribute to achievement of EMR and occurrence of imatinib‐related ADRs can help inform initial imatinib dose selection, identify patients that may benefit from an alternative TKI, and inform the use of therapeutic drug monitoring (TDM) to guide dose adjustments.

The aim of this study is to investigate predictions of imatinib steady‐state area under the concentration‐time curve (AUC_ss_), maximum concentration (C_ss,max_), C_ss,min_, and apparent clearance (CL/F) using a PBPK model in virtual patients with the demographic and clinical characteristics identical to those patients in the real‐world observational cohort study,^2^ and to investigate the association between predicted plasma exposure and clinical outcomes (severe ADRs, failure to achieve EMR). The PBPK model will also be used to provide mechanistic insight into factors contributing to variability in imatinib PK, and to explore the PK implications of concomitant medicines identified in the real‐world patient cohort study.

## 2 METHODS

**FIGURE 1:**
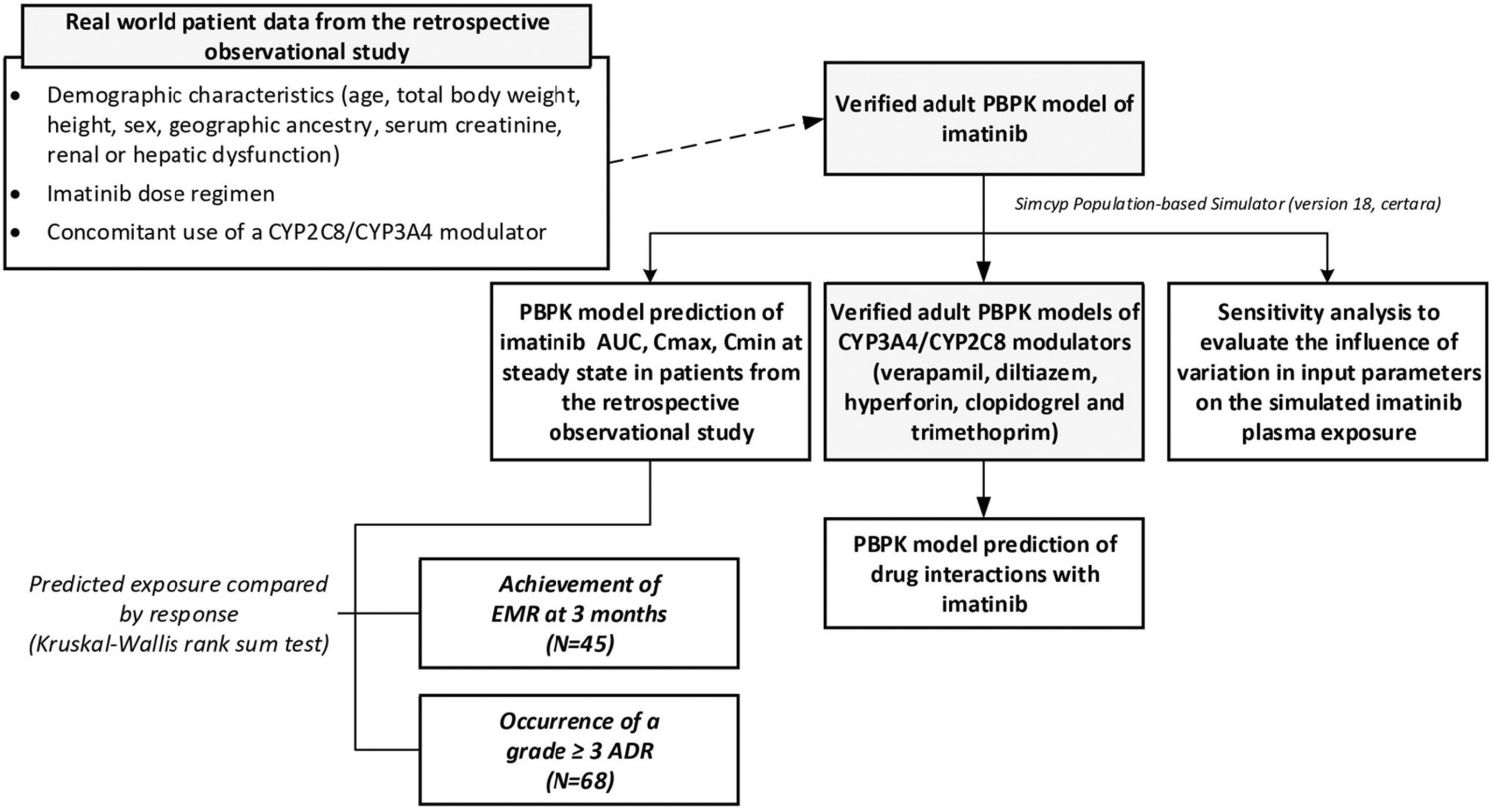
Schematic representation of the workflow of this study. ADR, adverse drug reaction; AUC, area under the plasma concentration‐time curve; C_max_, maximum (peak) plasma concentration; C_min_, minimum (trough) plasma concentration; CYP, cytochrome P450; EMR, early molecular response; PBPK, physiologically based pharmacokinetic.

A schematic representation of the study workflow is presented in Figure 1. All PBPK modeling and simulations were conducted using the Simcyp Population‐based Simulator (version 18, Certara UK Limited, Simcyp Division).^33^

### 2.1 Prediction of imatinib exposure in patients based on clinical outcomes

A previously developed and validated PBPK model of imatinib in adults^34^ was used to predict imatinib steady‐state systemic exposure in patients with CML from the real‐world retrospective observational study described in Adattini et al.^2^ The imatinib PBPK model has been verified extensively using clinical pharmacokinetic data from healthy and patient populations (gastrointestinal stromal tumor and CML) across different geographic ancestries and dosing regimens.^34^, ^35^ The imatinib PBPK model has also demonstrated excellent performance in predicting the drug interaction between imatinib (chronic use) and carbamazepine, a CYP3A4 and CYP2C8 inducer, with predicted pharmacokinetic parameters within 1.25‐fold of the clinically observed values.^34^

Details of the drug‐related input parameters from the imatinib PBPK model are listed in Supplementary Table 1. The fu_p_ of 0.05 for AAG binding was assigned to the PBPK model of imatinib based on the reported value in healthy Europeans.^36^, ^37^ Although higher plasma AAG concentrations have been reported in patients with solid tumors (1650 ± 160 mg L^−1^ in lung cancer; 1600 mg L^−1^ in breast cancer),^38^ plasma AAG concentrations reported in patients with chronic phase chronic myeloid leukemia (CML; 1200 ± 120 mg L^−1^ at diagnosis, and 1100 ± 60 mg L^−1^ at follow‐up)^39^, ^40^, ^41^ are closer to those reported in healthy individuals (mean 910 ± 40 mg L^−1^),^38^ corresponding to a similar unbound fraction in plasma (fu_p_) of imatinib in healthy individuals and patients with chronic phase CML.^41^ The corresponding dissociation constant was fixed and assumed to be independent of plasma AAG concentrations, with the variability in fu_p_ reflecting interindividual variability in AAG concentrations. The intrinsic clearances of imatinib to N‐desmethyl imatinib and other metabolites were estimated from in vitro kinetic data using recombinant CYP3A4 and human liver microsomes (HLM, in the presence of azamulin, a selective irreversible inhibitor of CYP3A4), with HLM (supplemented with azamulin) representing the CYP2C8 metabolic pathway given the assumed minor contribution (3%) of other CYP enzymes to imatinib metabolism.^42^ The mechanism‐based inhibition of CYP3A4 following multiple doses of imatinib was modeled by an increase in degradation of the active enzyme and thus, a decrease in enzyme activity over time, based on a turnover model detailed elsewhere.^43^ P‐gp and BCRP‐mediated biliary clearance of imatinib were modeled using data from previous in vitro studies.^44^, ^45^ The renal clearance (CL_R_) value for imatinib was taken from a study in patients with CML and Philadelphia chromosome‐positive acute lymphatic leukemia.^46^ Model assumptions are further detailed in Adiwidjaja et al.^34^ As the PBPK model of imatinib by Adiwidjaja et al^34^ used the Simcyp Simulator version 17 for model development and verification, the imatinib PBPK model was reverified using the Simcyp Simulator version 18 (Supplementary Methods, Supplementary Table 2).

Patient characteristics from the real‐world retrospective observational study^2^ were grouped according to achievement of the following clinical outcome measures:

1. Achievement of EMR (*BCR‐ABL1* ^*IS*^ ≤ 10%) at 3 months of imatinib treatment (as defined by the National Comprehensive Cancer Network^47^)
2. Occurrence of a grade ≥3 imatinib‐related ADR within the follow‐up period (defined using the Common Terminology Criteria for Adverse Events version 5 grading scale^48^)

In each scenario, individual patient parameters were added into the PBPK model for simulation of individual patient imatinib plasma concentration‐time profiles and estimation of AUC_ss_ (μg h mL^−1^), C_ss,max_ (μg mL^−1^), C_ss,min_ (μg mL^−1^) and CL/F (L h^−1^). Parameters used for virtual patients matched the real‐world patient cohort^2^ with respect to age (years), sex, actual body weight (ABW in kg; if available), height (cm; if available), serum creatinine concentration (μmol L^−1^; if available), hepatic function (defined by presence and severity of cirrhosis), kidney function (defined using glomerular filtration rate [GFR], estimated via the Chronic Kidney Disease – Epidemiology Collaboration equation^49^), geographic ancestry, imatinib dose (mg/day) and concomitant medicine use (if a validated model was available). The “Sim‐NEurCaucasian” population available in the Simcyp library was used for patients of European ancestry, and the “Sim‐Chinese”^50^ and “Sim‐Japanese”^51^ populations were used for patients of Chinese and Japanese ancestry, respectively. Details of the virtual populations used in the PBPK simulations, including for patients with hepatic or kidney dysfunction, are provided in the Supplementary Methods. PBPK model input variables of all patients from the real‐world observational study^2^ that were included in the PBPK simulations are presented in Supplementary Tables 3 and 4.

A total of 10 virtual trials for each simulation were carried out. A multiple‐dosing regimen of imatinib given for 14 days was used to achieve steady‐state. Potential differences in imatinib AUC_ss_, C_ss,max,_ C_ss,min_ and CL/F within different clinical scenario outcomes (i.e., between those who achieved EMR and those who did not) were evaluated by the independent two sample *t*‐test or the Kruskal‐Wallis rank sum test (if residuals were not normally distributed). A C_ss,min_ of 1002 ng mL^−1^ was the exposure target for clinical response used to interpret the simulated exposures, with previous studies correlating this threshold with a significantly higher probability of MMR achievement.^9^, ^52^ All reported *p* values are two‐sided, with a significance level of 0.05. All statistical analyses were conducted using R (version 3.3.3).^53^

### 2.2 Sensitivity analyses

Sensitivity analyses were conducted to evaluate the influence of variation in input parameters on the simulated imatinib plasma concentration at steady‐state.^33^, ^54^, ^55^ These analyses were conducted using either (a) the automated sensitivity analysis tool available in the Simcyp Simulator, or (b) repeated simulations using a trial design with virtual age and sex‐matched subjects, whilst adjusting values of the input parameter of interest (a total of 10 virtual trials for each subgroup within the input parameter, with 10 subjects per trial). Parameters selected for sensitivity analyses included sex, age, ABW, kidney or hepatic dysfunction (eGFR and cirrhosis, respectively), hepatic and intestinal CYP3A4 enzyme abundance, hepatic CYP2C8 enzyme abundance, hepatic P‐gp transporter abundance, hepatic BCRP transporter abundance, and AAG abundance.

To investigate the influence of variation in kidney function on imatinib exposure, default “Sim‐RenalGFR_less30” and “Sim‐RenalGFR_30to60” populations were used. Sensitivity analyses were also conducted using the modified kidney dysfunction populations (adjusted CYP2C8 and CYP3A4 abundances) described in Supplementary Methods. To investigate the influence of variation in hepatic function on imatinib exposure, default “Sim‐Cirrhosis‐CP” populations were used.^56^ The values for AAG abundance, drug‐metabolizing enzyme abundances, and transporter abundances used in the sensitivity analyses reflect physiologically plausible ranges. A meta‐analysis on the abundance of human hepatic CYP enzymes in Caucasian adult livers reported a CYP3A4 abundance of 93.0 pmol mg^−1^ protein (range, 0–600.0), and CYP2C8 abundance of 22.4 pmol mg^−1^ protein (range, 0–85.0), with a positive correlation between expression levels of CYP3A4 and CYP2C8 (Rs 0.68; *p <* 0.001).^57^ In healthy Caucasian adults, AAG concentration ranges from 0.36 to 1.46 g L^−1^.^58^ However, as an acute phase protein, AAG can increase approximately 2 to 6‐fold in many disease states, with concentrations up to 3.15 g L^−1^ reported.^22^ A meta‐analysis on the abundance of hepatic transporters in liver tissue of healthy Caucasian adults reported a weighted mean P‐gp abundance of 0.201 pmol per 10^6^ hepatocytes (CV 46%), and weighted mean BCRP abundance of 0.044 pmol per 10^6^ hepatocytes (CV 40%).^59^ Different types of liver damage have been shown to affect hepatocellular transport abundance to various extents.^59^, ^60^ Compared to healthy livers, hepatic P‐gp abundance was reported to increase up to 350% in cholestatic liver disease, and up to 410% in autoimmune hepatitis, with no significant differences observed in fatty liver disease, alcoholic liver disease, or hepatic virus C‐induced liver damage.^59^, ^60^ Conversely, hepatic BCRP abundance appears preserved in the these types of liver damage.^59^, ^60^ If examining hepatic transporter abundance according to the functional state of the liver, a Child‐Pugh class C score has been reported to result in a 260% increase in protein abundance of P‐gp, with no change in BCRP abundance.^60^

To determine whether model input parameters had statistically significant effects on imatinib PK parameters (AUC_ss,_ C_ss,max_, C_ss,min_ and CL/F), statistical analyses were conducted as described in the previous subsection. The parametric tests for comparing two (the independent two sample t‐test) or more groups (one‐way analysis of variance) were used. If the assumptions of normality and homogeneity of variances were not met, the corresponding non‐parametric tests (Wilcoxon‐Mann–Whitney and Kruskal‐Wallis rank sum test, respectively) were used.

### 2.3 Drug–drug interaction simulations

Imatinib is a substrate of CYP3A4 and CYP2C8,^13^ and has also been shown to inhibit its own CYP3A‐mediated metabolism after multiple‐dose administration.^42^ As such, PBPK simulations were performed to predict the extent of interaction between imatinib and CYP3A4/CYP2C8 modulators frequently used by patients in the real‐world retrospective observational study^2^; verapamil (a moderate CYP3A4 inhibitor), diltiazem (a moderate CYP3A4 inhibitor), trimethoprim (a weak CYP2C8 inhibitor), clopidogrel (moderate CYP2C8 inhibitor) and hyperforin (a CYP3A4 inducer in intestinal enzymes). The default PBPK model library files for verapamil and its metabolite norverapamil, diltiazem and its metabolite N‐desmethyldiltiazem, and trimethoprim were used as included in the Simcyp Simulator. The previously verified PBPK models of clopidogrel and its clopidogrel glucuronide metabolite^61^ were used to evaluate the potential CYP2C8 mediated drug–drug interaction with clopidogrel and imatinib. A verified PBPK model of hyperforin^62^ was used to predict the potential drug–drug interaction between St John's Wort and imatinib.

The simulations were performed using the default “Sim‐NEurCaucasian” population with a multiple‐dosing regimen of imatinib 400 mg daily for 14 days, with the trial design representative of the demographic characteristics of patients in the real‐world observational study (10 subjects, 4 females, age range 20–91 years).^2^ The CYP modulators were simulated at clinically relevant dose regimens for 14 days, except for trimethoprim which was dosed for 7 days to reflect clinical practice guidelines when treating urinary tract infections. A total of 10 virtual trials for each simulation were carried out to capture population variability. The interaction between imatinib and CYP modulators at steady‐state are reported as the geometric mean ratio of simulated PK parameters of imatinib (AUC_ss_, C_ss,max_, C_ss,min_ and CL/F) with and without the perpetrator drugs (CYP modulators), and their associated 90% confidence intervals. If the predicted ratio of PK parameters fell within 1.25‐fold (0.80–1.25), the interaction was not considered clinically significant.^63^

Key protein targets and ligands in this article are hyperlinked to corresponding entries in http://www.guidetopharmacology.org, the common portal for data from the IUPHAR/BPS Guide to PHARMACOLOGY,^64^ and  are permanently archived in the Concise Guide to PHARMACOLOGY 2019/20.^65^

## 3 RESULTS

### 3.1 Prediction of imatinib exposure in patients based on clinical outcomes

**FIGURE 2:**
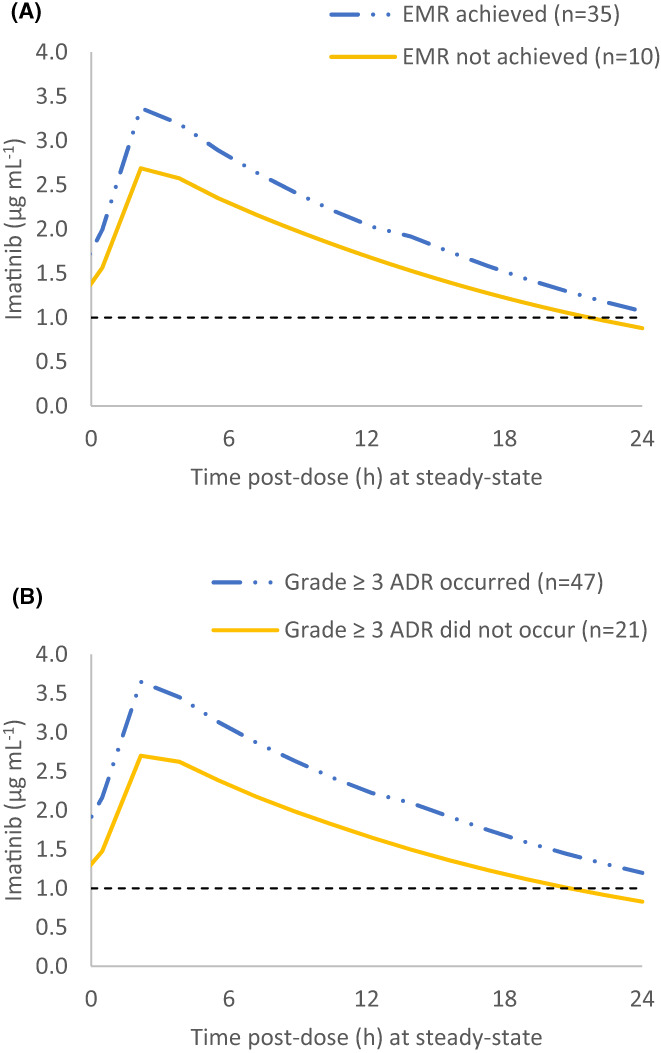
PBPK model simulated plasma concentration‐time profiles of imatinib in patients treated in the real‐world retrospective observational study,^2^ grouped by clinical outcome; (A) achievement of EMR at 3 months (N = 45), (B) occurrence of a grade ≥3 imatinib‐related ADR (*N* = 68). Geometric mean simulated plasma concentration time curves of imatinib at steady‐state in patients who experienced (blue dotted line) and did not experience (orange line) the clinical outcome of interest in the retrospective observational study are presented. The C_ss,min_ target of 1.002 μg mL^−1^ is shown by dashed black lines. ADR, adverse drug reaction; C_ss,min_, minimum (trough) plasma concentration at steady‐state; EMR, early molecular response at 3 months; PBPK, physiologically based pharmacokinetic.

**FIGURE 3:**
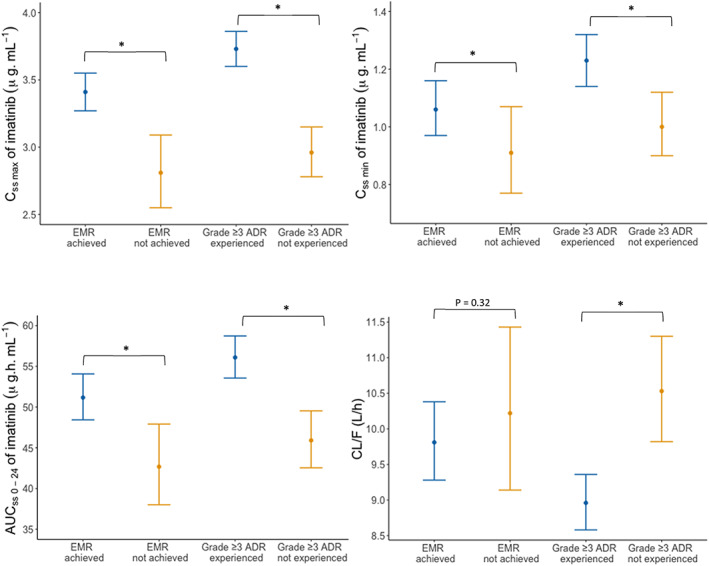
PBPK model simulated PK parameters of imatinib in patients treated in the real‐world retrospective observational study,^2^ grouped by achievement of EMR or occurrence of a grade ≥3 imatinib‐related ADR. Simulation results are depicted as geometric mean predicted C_ss,max_, C_ss,min_, AUC_0‐24,ss_ and CL/F of imatinib (and 95% confidence intervals) in patients who experienced the clinical outcome of interest (blue line) and in patients who did not experience the clinical outcome of interest (orange line) in the retrospective observational study. 35 patients achieved EMR, whilst 10 did not achieve EMR. 47 patients experienced a grade ≥3 ADR, whilst 21 did not. *Statistically significant difference (*p* < 0.05; Kruskal‐Wallis rank sum test). ADR, adverse drug reaction; AUC_0‐24,ss_, area under the plasma concentration‐time curve from time zero to 24 h after a dose at steady‐state; C_ss,max_, maximum (peak) plasma concentration at steady‐state; C_ss,min_, minimum (trough) plasma concentration at steady‐state; CL/F, apparent clearance; EMR, early molecular response at 3 months; PBPK, physiologically based pharmacokinetic; PK, pharmacokinetic.

Individual patient characteristics, imatinib dose regimens and simulated PK parameters are presented in Supplementary Tables 3 and 4. The simulated geometric mean plasma concentration‐time profiles of imatinib at steady‐state for different clinical scenarios are summarized in Table 1 and Figures 2 and 3. Notably, the simulated geometric mean AUC during 24 h after the dose (AUC_0‐24,ss_), C_ss,max_ and C_ss,min_ of imatinib were significantly higher in patients who achieved EMR at 3 months, compared to those who did not (p < 0.05). Unlike patients who did not achieve EMR, the geometric mean C_ss,min_ of patients who achieved EMR fell above the efficacy target of 1002 ng mL^−1^.^9^, ^52^ Moreover, imatinib AUC_0‐24,ss_, C_ss,max_, and C_ss,min_ were significantly higher in patients who experienced a grade ≥3 ADR on imatinib treatment compared to those who did not (p < 0.05).

**TABLE 1:** Summary of PBPK model predicted PK parameters of imatinib following multiple‐dose regimens, in patients with CML followed in the real‐world retrospective observational study,^2^ grouped by clinical scenario outcome.

PBPK parameter prediction	EMR achieved (*n* = 35)	EMR not achieved (*n* = 10)	Significance of predicted EMR difference	Grade ≥3 ADR experienced (*n* = 47)	Grade ≥3 ADR not experienced (*n* = 21)	Significance of predicted ADR difference
AUC_0‐24,ss_ (μg h mL^−1^)	51.17 (48.43—54.07)	42.68 (38.01—47.91)	*p* < 0.001*	56.09 (53.56—58.73)	45.91 (42.54—49.54)	*p* < 0.001*
C_ss,min_ (μg mL^−1^)	1.06 (0.97—1.16)	0.91 (0.77—1.07)	*p* < 0.05*	1.23 (1.14—1.32)	1.00 (0.90—1.12)	*p* < 0.05*
C_ss,max_ (μg mL^−1^)	3.41 (3.27—3.55)	2.81 (2.55—3.09)	*p* < 0.001*	3.73 (3.60—3.86)	2.96 (2.78—3.15)	*p* < 0.001*
CL/F (L h^−1^)	9.81 (9.28—10.38)	10.22 (9.14—11.43)	*p* = 0.32	8.96 (8.58—9.36)	10.53 (9.82—11.30)	*p* < 0.001*

Notes. A total of 10 trials for each simulation were carried out. All pharmacokinetic parameters are reported as geometric mean values (95% confidence intervals) of PBPK model predictions.

Abbreviations: ADR, adverse drug reaction; AUC_0‐24,ss_, area under the plasma concentration‐time curve from time zero to 24 h after a dose at steady‐state; C_ss,max_, maximum (peak) plasma concentration at steady‐state; C_ss,min_, minimum (trough) plasma concentration at steady‐state; CL/F, apparent clearance; CML, chronic myeloid leukemia; EMR, early molecular response at 3 months; PBPK, physiologically based pharmacokinetic; PK, pharmacokinetic.

^*^ Statistical significance evaluated using Kruskal‐Wallis Rank Sum Test.

### 3.2 Sensitivity analyses

The impact of various parameters on simulated imatinib systemic exposure is shown in Supplementary Figure 1. The simulated plasma concentration‐time profile of imatinib was sensitive to variation in subject age, in which a change in age from 20 to 55 years increased imatinib AUC_0‐24,ss_ by 30% (p < 0.05) and increased C_ss,min_ by 46% (p < 0.05). The effect was notably larger in older age, with a change in age from 55 to 90 years increasing imatinib AUC_0‐24,ss_ and C_ss,min_ 2‐fold and 2.6‐fold, respectively (p < 0.05). Increased ABW (45–125 kg) was associated with significantly decreased imatinib AUC _0‐24,ss_ (44%) and C_ss,min_ (40%, *p* < 0.05). Although C_ss,max_ was modestly higher in females (18%; *p <* 0.05), other PK parameters were not significantly altered by sex.

Variation in hepatic CYP2C8 and CYP3A4 enzyme abundances, and in AAG concentrations, significantly influenced imatinib systemic exposure. A hepatic CYP2C8 abundance range of 0–85 pmol mg^−1^ protein decreased imatinib AUC_0‐24,ss_ and C_ss,min_ by 70% and 85%, respectively (*p* < 0.05). A hepatic CYP3A4 enzyme abundance range of 0 to 600 pmol mg^−1^ protein decreased imatinib AUC_0‐24,ss_ and C_ss,min_ by 50% and 68%, respectively (*p* < 0.05). As AAG concentration increased from 0.3 to 3.2 mg/mL, total plasma imatinib AUC_0‐24,ss_ increased by 8.4‐fold (*p* < 0.05) and C_ss,min_ increased by 26‐fold (*p* < 0.05). Intestinal CYP3A4 enzyme abundance, and hepatobiliary P‐gp and BCRP transporter abundances, had no significant impact on simulated imatinib exposure.

Simulated imatinib plasma concentration‐time profiles were sensitive to varying degrees of hepatic impairment (measured as liver cirrhosis) and kidney dysfunction (measured as eGFR). Child‐Pugh B cirrhosis was associated with a 36% increase in imatinib AUC_0‐24,ss_ and 56% increase in C_ss,min_ compared to healthy (“Sim‐NEurCaucasian”) adults (*p* < 0.05), whilst Child‐Pugh C cirrhosis was associated with a 66% increase in AUC_0‐24,ss_ and 100% increase in C_ss,min_ relative to healthy adults (*p* < 0.05). Child‐Pugh A cirrhosis did not significantly alter simulated imatinib exposure. In the default kidney dysfunction simulations, a GFR of 30–60 mL/min/1.73 m^2^ was associated with a 53% higher AUC_0‐24,ss_ and 85% higher C_ss,min_ relative to healthy adults (*p* < 0.05), whilst a GFR < 30 mL/min/1.73 m^2^ was associated with a 2.6‐fold increase in AUC_0‐24,ss_ and 3.4‐fold increase in C_ss,min_ relative to healthy adults (*p* < 0.05). Simulated imatinib systemic exposure remained sensitive to kidney dysfunction in the modified populations (CYP enzyme abundance changed to default values in general populations), however, to a lesser extent (AUC_0‐24,ss_ and C_ss,min_ increased by 1.75 and 2.3‐fold, respectively, for GFR < 30 mL/min/1.73 m^2^).

### 3.3 Drug–drug interaction simulations

**FIGURE 4:**
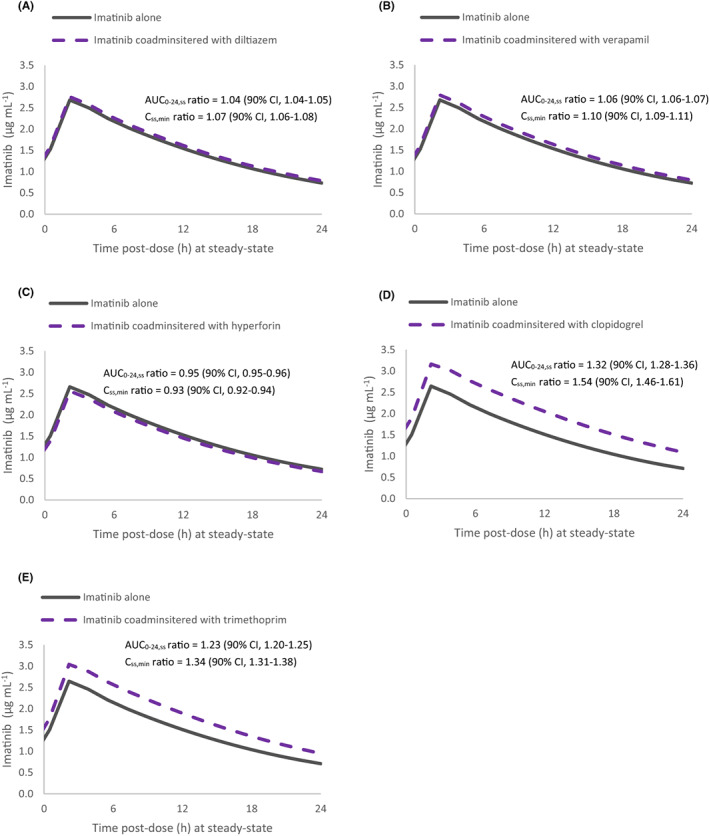
PBPK model prediction of the interactions between imatinib and a set of CYP modulators at steady‐state (A) diltiazem 45 mg QID for 14 days, (B) verapamil 60 mg TDS for 14 days, (C) hyperforin (St John's Wort) 45 mg daily for 14 days, (D) clopidogrel 75 mg daily for 14 days, (E) trimethoprim 300 mg daily for 7 days (day 8–14). Imatinib was administered at 400 mg daily for 14 days in a North European Caucasian population, with 10 subjects (4 females) and an age range of 20–91 years. Simulation results are depicted as geometric mean predicted plasma concentrations of imatinib in the presence and absence of coadministration with the CYP modulator. The extent of interactions was evaluated based on predicted pharmacokinetic parameter ratios (ratio in the presence and absence of CYP modulators) and their 90% confidence intervals (CI). A ratio of 1 indicates absence of drug interactions with imatinib. AUC_0‐24,ss_, area under the plasma concentration‐time curve from time zero to 24 h after a dose at steady‐state; C_ss,min_, minimum (trough) plasma concentration at steady‐state; CYP, cytochrome P450; PBPK, physiologically based pharmacokinetic; QID, four times a day; TDS, three times a day.

The results of the simulations to predict the interactions between selected CYP modulators and imatinib are detailed in Figure 4 and Supplementary Table 5. Notably, the AUC_0‐24,ss_ and C_ss,min_ of imatinib were predicted to increase by 23% and 34%, respectively, in the presence of trimethoprim. In the presence of clopidogrel, the AUC_0‐24,ss_ of imatinib was simulated to increase by 32% and C_ss,min_ predicted to increase by 54%. Meanwhile, the interactions between imatinib and diltiazem (AUC_0‐24,ss_ ratio = 1.04), and between imatinib and verapamil (AUC_0‐24,ss_ ratio = 1.06) were predicted to be non‐clinically significant at steady‐state. At the recommended dosing regimen of St John's Wort (hyperforin 45 mg daily), there was minimal interaction with imatinib at steady‐state (AUC_0‐24,ss_ ratio = 0.95; C_ss,max_ ratio = 0.96; C_ss,min_ ratio = 0.93; CL/F ratio = 1.05). This mirrors the geometric mean ratios reported in the PBPK drug–drug interaction simulations of imatinib with hyperforin by Adiwidjaja et al^62^ (AUC_0‐24,ss_ ratio = 0.94; C_ss,max_ ratio = 0.95).

## 4 DISCUSSION

This study utilized a PBPK approach to predict the imatinib plasma concentration‐time profile in patients with CML included in a real‐world study to investigate whether imatinib systemic exposure differed for patients who achieved EMR or experienced severe ADRs. The PBPK model predicted significantly higher imatinib AUC_ss_, C_ss,max_ and C_ss,min_ in patients who achieved EMR at 3 months, compared to patients who did not. This relationship between predicted imatinib systemic exposure and EMR achievement is a key finding of this analysis and has not been previously reported in the literature. Previous studies have described the relationship between imatinib systemic exposure and other clinical response measures. A population pharmacokinetic analysis of imatinib in a European cohort (*n* = 20) demonstrated a significant correlation between lower imatinib unbound clearance and achievement of hematological response (Odds ratio [OR], 0.8 ± standard error 0.9; *p* < 0.05).^66^ Many studies have correlated imatinib C_ss,min_ with complete cytogenetic response (CCyR) and MMR achievement.^8^, ^9^, ^11^, ^52^, ^67^, ^68^, ^69^, ^70^, ^71^, ^72^, ^73^ In a European cohort study of patients treated with imatinib for at least 12 months (*n* = 68), mean (± standard deviation [SD]) C_ss,min_ was significantly higher in those who had achieved CCyR than in those who had not (1123 ± 617 ng mL^−1^ vs. 694 ± 556 ng mL^−1^, respectively; *p* < 0.05), and again higher in the group that had achieved MMR than in the group who had not (1452 ± 649 ng mL^−1^ vs. 869 ± 427 ng mL^−1^, respectively; *p* < 0.001).^9^ A pharmacokinetic analysis of the TOPS study (*n* = 240) showed significantly faster times to MMR, and higher rates of both MMR and CCyR at 6 and 12 months, when C_ss,min_ ≥ 1165 ng mL^−1^.^67^ Imatinib C_ss,min_ > 1000 ng mL^−1^ predicted improved MMR, event‐free and failure‐free survival in a cohort of Chinese patients with CML (*n* = 110), with those who achieved MMR showing higher mean imatinib C_ss,min_ compared with those not achieving MMR (1474 ± SD 419 ng mL^−1^ vs. 986 ± 213 ng mL^−1^, respectively; *p* < 0.001).^73^ A study in Japanese patients with CML treated with imatinib (*n* = 254) observed a significantly lower probability of achieving MMR in patients with C_ss,min_ < 1002 ng mL^−1^.^52^

Another major finding from these PBPK simulations was the significantly higher mean simulated imatinib systemic exposure in CML patients who experienced a grade ≥3 imatinib‐related ADR, compared to patients who did not. Previous studies have correlated imatinib plasma concentration with toxicity^8^, ^66^, ^67^, ^74^ however, a consistent threshold concentration has not been observed. In a sub‐analysis of the IRIS study (*n* = 351), fluid retention, rash, myalgia, and anemia were more common in patients who demonstrated higher plasma imatinib concentrations (C_ss,min_ > 1170 ng mL^−1^) compared to those with lower concentrations (C_ss,min_ ≤ 1170 ng mL^−1^).^8^ A population pharmacokinetic model (*n* = 20) demonstrated that unbound imatinib AUC was significantly correlated with the occurrence and number of side effects (OR, 6.1 ± standard error 4.5; *p* < 0.05).^66^ A cohort study in South Asian patients (*n* = 111) demonstrated a higher C_ss,min_ in patients who experienced grade ≥2 thrombocytopaenia compared to those who did not experience thrombocytopaenia (C_ss,min_ 1582 ± SD 355 ng mL^−1^ vs. 1337 ± 366 ng mL^−1^, respectively; *p* < 0.05).^74^ Imatinib C_ss,min_ > 1000 ng mL^−1^ was correlated with leukopenia or neutropenia, diarrhea, periorbital and limb oedema, and rash (*p* < 0.05) in a Chinese cohort (*n* = 110), with an AUC_ss_ threshold of 1685 ng mL^−1^ identified for diarrhea and 1575 ng mL^−1^ for periorbital and limb oedema.^73^ The TOPS study (*n* = 240) noted a significantly higher incidence of all‐grade neutropenia, anemia, leukopaenia, rash, diarrhea, arthralgia/myalgia and all‐cause oedema in patients with a C_ss,min_ > 3180 ng mL^−1^.^67^ Further research is required to define the concentration‐threshold for imatinib‐related ADRs, which may help facilitate TDM‐guided dose adjustments and improve tolerability outcomes.

The predicted exposures for the various treatment outcome groups were a consequence of the differences in patient and medication‐related factors, and relationships between these covariates, that could be accommodated within the PBPK modeling and simulation software. Sensitivity analyses provided mechanistic insight into the factors contributing to inter‐individual variability in imatinib PK. The predicted imatinib plasma concentration–time profile was sensitive to changes in ABW, in line with previous studies where higher ABW was correlated to increased imatinib apparent volume of distribution (V/F) and CL/F,^75^, ^76^, ^77^, ^78^ and lower C_ss,min_.^8^, ^67^ Another body size metric, body surface area (BSA), has been demonstrated as an important covariate of imatinib PK variability.^8^, ^67^, ^79^ Accordingly, in both univariable and multivariable analyses in the retrospective observational study,^2^ lower ABW was associated with higher rates of occurrence of grade ≥3 ADR during imatinib treatment.

Sensitivity analysis identified age as another influential factor contributing to variability in imatinib PK. Older age was associated with reduced imatinib CL/F and increased C_ss,min_, C_ss,max_ and AUC_ss_, confirming results from other PK studies.^8^, ^80^ The effect of age on imatinib PK supports the findings of the retrospective observational study,^2^ whereby older age was associated with superior sDMR rates in univariable regression, and a higher Charlson Comorbidity Index score (of which age is included as a predictor) was associated with increased grade ≥3 ADRs in both univariable and multivariable regression.^2^

Interestingly, sex was identified as an influential factor in the variability of imatinib C_ss,max_, with no significant influence on other PK parameters. Previous studies in patients with CML have observed significantly higher imatinib C_ss,min_ in females compared to males, with authors highlighting differences in body weight and BSA as potential contributors.^67^, ^81^, ^82^ Regression analysis from the observational study presented in Adattini et al^2^ identified female sex as independently predictive of recurrent imatinib‐related grade ≥3 ADRs, which could be attributed to lower ABW (median 64.5 vs. 82 kg in males, *p* < 0.001). Although not accounted for in these PBPK simulations, sex differences in expression levels of hepatic P‐gp transporters may also play a role in altered imatinib PK,^59^ however, the evidence is conflicting.^83^

In line with previous studies, the simulated imatinib plasma concentration was sensitive to changes in AAG abundance. A reduction in AAG concentration leads to an increase in the unbound fraction of imatinib and a reduction in total plasma imatinib concentrations, due to the rapid distribution of unbound imatinib into extravascular space and increased total clearance.^77^, ^84^, ^85^, ^86^, ^87^, ^88^, ^89^, ^90^ Elevated AAG concentrations in patients have also been correlated with delayed response or resistance to imatinib treatment in CML, with studies suggesting that higher AAG concentrations are associated with reduced distribution into leukemic cells.^23^, ^86^, ^88^ As discussed previously, AAG demonstrates highly variable concentrations, increasing 2‐ to 6‐fold in response to injury, infection, inflammation, and cancer.^22^, ^23^ Males have significantly higher mean AAG concentrations than females.^91^ There are also inter‐ethnic differences in AAG concentrations, with individuals of Asian ancestry reported to have lower AAG concentrations than Europeans.^91^, ^92^, ^93^ Additionally, drug–drug interactions can influence protein binding and subsequently the unbound concentration of a drug, as observed in interaction between clindamycin and imatinib.^85^

Liver dysfunction was identified in these PBPK simulations as influential on imatinib PK, with increasing severities of liver dysfunction associated with increased imatinib systemic exposure. Similarly, Lankheet et al^10^ observed a tendency toward higher imatinib plasma concentrations with decreasing liver function. A higher rate of imatinib‐related haematologic toxicities and more frequent dose reductions have also been observed in patients with pre‐existing liver dysfunction.^94^ The decline in activity of CYP enzymes in enterocytes and hepatocytes is more pronounced as the severity of cirrhosis increases,^95^ explaining the different magnitude of effects simulated on imatinib PK in patients with Child‐Pugh B/C cirrhosis and Child‐Pugh A cirrhosis. In advanced cirrhosis, kidney dysfunction is often also present, thereby reducing renal elimination.^96^ Aside from PK changes, patients with cirrhosis can exhibit different pharmacological and toxicological responses to imatinib due to other pathophysiological changes.^96^

The observed influence of kidney dysfunction on the PK variability of imatinib is supported in previous studies. A study by the National Cancer Institute Organ Dysfunction Working Group reported significantly higher imatinib exposure (dose‐normalized AUC_0‐24,ss_ and C_ss,max_), lower V/F and CL/F, and higher incidences of serious ADRs in patients with a creatinine clearance (CrCL) < 60 mL/min compared to normal kidney function.^88^ Analogous to these findings, Tong et al^94^ reported a higher rate of imatinib‐related hematological toxicities and more frequent dose reductions in patients with pre‐existing kidney dysfunction (CrCL <60 mL/min) compared to normal kidney function. Only a relatively small fraction of imatinib is excreted unchanged in the urine,^97^ and hence decreased imatinib clearance in patients with kidney dysfunction is likely to be associated with altered tissue binding and plasma protein binding, the latter of which was accounted for in the virtual population with renal impairment during PBPK simulations by higher concentrations of plasma AAG.^98^, ^99^

Geographic ancestry is another factor that can contribute to inter‐individual variability in imatinib PK and outcomes, due to ethnic variations in the expression or activity of genes encoding for drug‐metabolizing enzymes, plasma protein binding and drug transporters, ethnic variation in complementary and herbal medicine use and dietary habits, and ethnic variation in body composition.^18^ A Japanese study (*n* = 31) observed a mean C_ss,min_ of 1400 ± 570 ng mL^−1^ with imatinib 400 mg/day, and demonstrated that a lower imatinib dose of 300 mg/day was successful in maintaining a C_ss,min_ above the threshold of 1002 ng mL^−1^ (mean C_ss,min_ of 1150 ± SD 440 ng mL^−1^).^100^ Similarly, a lower imatinib dose of 300 mg/day in a Chinese cohort (*n* = 16) was successful in maintaining concentrations required for MMR achievement, with a mean imatinib C_ss,min_ of 1034 ng mL^−1^ ± 576 ng mL^−1^.^73^ European cohorts have reported lower systemic exposures with imatinib 400 mg/day, with an overall mean C_ss,min_ of 979 ± 530 ng mL^−1^ in the IRIS trial^8^ and 1058 ± 557 ng mL^−1^ in a French study.^9^ Interestingly, our retrospective study observed a lower hazard of recurrent imatinib‐related grade ≥3 ADRs in patients of European ancestry compared to East Asian ancestry.^2^

In this study, a PBPK modeling approach was utilized to evaluate drug–drug interactions with imatinib and CYP modulators at steady‐state. The predicted drug–drug interaction effects between imatinib and verapamil, diltiazem and hyperforin (St John's Wort) at steady‐state were not clinically significant. Verapamil and diltiazem are inhibitors of CYP3A4 (via a mechanism‐based inhibition) and P‐gp, whilst hyperforin is an inducer of CYP3A4, CYP2C9, CYP2C19 and P‐gp. Repeat‐dose administration of imatinib results in autoinhibition of CYP3A4‐mediated metabolism, assigning a key role to other biotransformation pathways such as CYP2C8, and diminishing the effect of CYP3A4 modulators on imatinib at steady‐state.^42^, ^101^ In agreement with this, sensitivity analysis in this study demonstrated a negligible change in imatinib systemic exposure at steady‐state when CYP3A4 enzyme abundance in hepatocytes dropped from 93 pmol mg^−1^ protein (mean abundance in healthy Caucasian livers^57^) to 0 pmol mg^−1^ protein (analogous to mechanism‐based inhibition of hepatic CYP3A4). Conversely, at steady‐state, imatinib is predicted to be more susceptible to drug interactions with inducers of hepatic CYP3A4. Significant decreases of imatinib C_ss,min_ have been observed in patients receiving CYP3A4 enzyme‐inducing anti‐epileptic drugs, such as carbamazepine and phenytoin.^10^, ^102^ However, since imatinib undergoes little to no metabolism in enterocytes,^62^ it is unlikely that CYP3A inducers confined to intestinal enzymes (e.g., hyperforin) will affect the steady‐state CL/F of imatinib. This is reflected in the sensitivity analysis of CYP3A4 in this study, whereby a change in CYP3A4 enzyme abundance in enterocytes resulted in negligible changes to imatinib systemic exposure.

A PBPK‐based analysis provided a prediction of imatinib drug–drug interactions with CYP2C8 inhibitors, with simulations of repeat dosing of trimethoprim and clopidogrel (and its glucuronide metabolite) increasing the AUC_ss_ of imatinib by 23% and 32%, respectively. Clinical drug–drug interaction data with CYP2C8 inhibitors and imatinib at steady‐state is lacking, however, a candidate‐gene study of CYP2C8 supports the clinically significant effect of reduced CYP2C8 function on imatinib C_ss,min_.^12^ Whilst the dosing regimen and potency of the inhibitor determine the extent to which it affects systemic exposure of the victim drug, recent studies have demonstrated that genomic data may be used to estimate the magnitude of potential drug–drug interactions (and vice versa).^103^, ^104^ CYP2C8 inducers may also influence imatinib disposition, however clinical PK data are limited. Accounting for CYP2C8 induction in a PBPK model of carbamazepine (and its active metabolite carbamazepine‐10,11‐epoxide) improved the prediction of its interaction with imatinib at steady‐state,^34^ highlighting the importance of accounting for CYP2C8‐based interactions with imatinib. Together with in vitro and in silico studies,^34^, ^42^, ^62^, ^101^, ^103^ these simulations suggest that pharmacogenetic polymorphisms and drug–drug interactions affecting CYP2C8 activity may be another confounding factor on the total plasma imatinib concentration‐response relationship. CYP2C8 inhibition could be the mechanism behind the superior molecular response rates observed in patients treated with imatinib on concomitant potentially interacting medicines in the retrospective study.^2^

TDM has been advocated as an integral part for dose optimisation of imatinib, given the high inter‐patient PK variability and established relationship between exposure and drug response (pharmacological effect and treatment‐related toxicities).^73^, ^105^, ^106^ The PBPK modeling approach may help guide dose selection of imatinib for individual patients based on patient characteristics and has an advantage over the population pharmacokinetic counterpart in predicting the effect of comedications, particularly for previously unstudied/unidentified clinical interactions. Implementing PBPK model‐informed precision medicine in clinical practice involves the concept of matching the attributes of the real patient to those of his or her ‘virtual twin’, informed by covariates known to influence the drugs pharmacokinetics.^107^, ^108^ This approach may decrease variability in drug exposure via individualized dose adjustment.^108^ Although the PBPK model of imatinib^34^ may be useful to guide dose selection and optimisation of imatinib in real‐world clinical practice, further prospective PBPK model‐informed TDM studies are desirable to evaluate the robustness of the model.

A limitation of this research is that the PBPK model of imatinib used in these simulations did not include transporter mediated process in the apical/luminal membrane of enterocytes.^34^ Therefore, there is a possibility that the drug–drug interaction simulations could have underestimated imatinib plasma‐concentration in the presence of P‐gp inhibitors. Another limitation is the inability to predict intracellular concentrations of imatinib. Imatinib has an intracellular site of action, therefore variability in imatinib disposition into target CML cells may have important implications on imatinib response.^7^ In vitro studies have demonstrated reduced intracellular concentrations of imatinib, and reduced phosphorylation of the BCR‐ABL1 downstream effector phospho‐CRKL, in *BCR‐ABL1* positive and P‐gp positive cells—a possible mechanism of imatinib resistance.^14^, ^15^, ^16^, ^17^ Further research is required to investigate the CML cell intracellular concentration‐response relationship. Another limitation of this study is that potential non‐adherence to imatinib treatment could not be accounted for. An assumption was made that patients took the prescribed dose 100% of the time leading up to the outcome of interest. Due to the prolonged (potentially life‐long) course of CML treatment, adherence is critical to achieving and maintaining adequate response. There is a clear correlation between adherence to TKI treatment and achieving optimal efficacy outcomes.^109^, ^110^, ^111^, ^112^ Studies in patients with CML reported as few as 14% to 33% of patients were highly adherent to their TKI treatment.^109^, ^113^, ^114^, ^115^ Therefore, poor adherence could be an additional factor contributing to the lack of achievement of molecular response outcomes in the retrospective observational study.^2^ Furthermore, due to the lack of clinical drug–drug interaction studies of imatinib with trimethoprim, clopidogrel, verapamil and diltiazem, the PBPK simulated drug–drug interactions could not be verified with clinically observed concentrations. Further clinical studies are required to confirm the drug–drug interactions simulated in this study.

This study demonstrates the utility of PBPK modeling in providing a mechanistic framework that can extend our understanding of the inter‐individual variability in treatment outcomes of patients in routine daily clinical practice. We present new insights on the relationship between imatinib systemic exposure and imatinib treatment outcomes, and identify patient and medication‐related factors that could influence imatinib systemic exposure in real‐world populations. The outcomes of this study support the rationale for TDM to guide imatinib dosing to achieve optimal outcomes in patients with CML.

## AUTHOR CONTRIBUTIONS

All authors conceived the study and contributed to the interpretation. J.A.A performed the simulation, analyzed the data and drafted the manuscript. J.A, A.S.G and A.J.M critically revised the manuscript. All authors approved the final manuscript.

## FUNDING INFORMATION

This work was supported by the Peter Coates Postgraduate Scholarship in Ethnopharmacology provided by GlaxoSmithKline.

## CONFLICT OF INTEREST STATEMENT

The authors declare that the research was conducted in the absence of any commercial or financial relationships that could be construed as a potential conflict of interest.

## ETHICS STATEMENT

The real world clinical data included in this study was collected with the approval of the Sydney Local Health District Human Research Ethics Committee (reference number: LNR/17/CRGH/248).

## Supporting information


**Data S1:** Supporting Information

## Data Availability

The data that support the findings of this study are available on request from the corresponding author. The data are not publicly available because of privacy or ethical restrictions.
