# Supplementary material for: Application of physiologically based pharmacokinetic modeling to understand real‐world outcomes in patients receiving imatinib for chronic myeloid leukemia
Source: Pharmacol Res Perspect. 2023 Jul 7;11(4):e01082. doi: 10.1002/prp2.1082 (PMC10326685; doi:10.1002/prp2.1082)
Supplement: Supplementary file 1 — Data S1: Supporting Information [file PRP2-11-e01082-s001.docx]

ONLINE SUPPLEMENTARY MATERIAL

**Application of physiologically-based pharmacokinetic modelling to understand real-world outcomes in patients receiving imatinib for chronic myeloid leukaemia**

Josephine A Adattini, Jeffry Adiwidjaja, Annette S Gross, Andrew J McLachlan

SUPPLEMENTARY METHODS

Verification of previously developed physiologically-based pharmacokinetic modelling (PBPK) model using Simcyp Simulator version 18

The PBPK model of imatinib by Adiwidjaja et al^1^ used the Simcyp Simulator version 17 for model development and verification. Therefore, the imatinib PBPK model was reverified using the Simcyp Simulator version 18. PBPK simulations were performed with the number of people, proportion of males/females, age range, and dosing regimens matched to the corresponding published clinical studies with pharmacokinetic (PK) data. Only clinical studies with multiple dosing regimens were considered. A total of 10 virtual trials for each simulation were carried out.

A comparison of the PK parameters obtained from the simulation with those observed from clinical studies are presented in Supplementary Table 2. Prediction differences of imatinib PK parameters at steady-state are expressed as the ratio of the Simcyp Simulator (version 18) PBPK model prediction to the clinically reported parameter value. All key PK parameters of imatinib were predicted within a 1.25-fold difference of the clinically reported parameters (range, 1.02 – 1.14), indicating that the imatinib PBPK model adequately performs in predicting observed clinical PK data in version 18 of the Simcyp Simulator. Simulations were repeated with modifications to the α_1_-acid glycoprotein (AAG) abundance in Simcyp Simulator (version 18) to reflect those in Simcyp Simulator (version 17); males 0.81 g L^-1^ (Coefficient of variation [CV] 15%) and females 0.79 g L^-1^ (CV 13%). The predicted PK parameters using the AAG modified Simcyp Simulator version 18 were also within 1.25-fold (range, 0.93 – 1.08) of the clinically reported parameters.

Details of the model input parameters and virtual populations used in the PBPK simulations

Age, actual body weight (ABW) and serum creatinine concentration at the time of occurrence of a grade ≥ 3 imatinib-related adverse drug reaction (ADR) in the real-world retrospective observational study^2^ were used in the simulations. If a grade ≥ 3 ADR was not experienced during imatinib treatment, age, ABW and serum creatinine concentration at the end of study follow-up were used in the simulations. For simulations of Early Molecular Response (EMR) achievement, an individual’s age, ABW and serum creatinine concentration at 3 months of imatinib treatment were used. In cases of unknown ABW, the population median was used.

The dosing regimen of imatinib that the patient was using in the 14 days leading up to the event of interest^2^ was used for simulations. If the patient did not experience a grade ≥ 3 ADR, the dose regimen used in the simulation was the last recorded dose at the end of the study follow-up period. If the patient did not achieve EMR at 3 months, the last recorded dose at 3 months was used in the simulation. Concomitant medicines with the potential to modulate cytochrome P450 (CYP)3A4 and/or CYP2C8, that were used by patients leading up to the event of interest, were incorporated into the respective simulations if a PBPK model was available. Patients using other potentially interacting medicines without available validated PBPK models (i.e., black cohosh, milk thistle, voriconazole) were excluded from simulations. PBPK simulations for the CYP modulators were carried out using the dosing regimens observed in the real-world observational study.

The general adult populations available in the Simcyp platform were used for all simulations. Although Simcyp Simulator includes an *in silico* cancer population (“Sim-Cancer”), it comprises data from North European Caucasian individuals with solid tumours. The “Sim-Cancer” model also has a correction to account for the increased plasma volume, reduced levels of albumin, increased levels of AAG, and changes in transporter abundance, but not of CYP enzymes in patients with solid tumours. However, plasma AAG concentrations reported in healthy individuals (mean 910 ± 40 mg L^-1^)^3^ are closer to those reported in patients with chronic phase chronic myeloid leukaemia (CML; 1,200 ± 120 mg L^-1^ at diagnosis, and 1,100 ± 60 mg L^-1^ at follow-up),^4-6^ compared to patients with solid tumours (1,650 ± 160 mg L^-1^ in lung cancer; 1,600 mg L^-1^ in breast cancer).^3^ This corresponds to a similar unbound fraction in plasma (fu_p_) of imatinib in healthy individuals (fu_p_ of 0.05, range 0.03 to 0.10)^7^ and patients with chronic phase CML (fu_p_ of 0.03, range 0.01 to 0.10).^6^ As the physiology of a patient with chronic phase CML is closer to the general population than individuals living with a solid tumour, the general adult populations available in the Simcyp library were used in this study (e.g., “Sim-NEurCaucasian”, “Sim-Chinese”,^8^ and “Sim-Japanese”^9^).

In patients with hepatic impairment, default “Sim-Cirrhosis-CP” populations^10^ available in the Simcyp Simulator were used. The “Sim-Cirrhosis-CP” populations incorporate information on hepatic blood flow, CYP enzymes, liver size, plasma protein binding and kidney function in the virtual population libraries, to correspond to Child-Pugh scores A (mild), B (moderate) and C (severe) liver cirrhosis.^10^

Default “Sim-RenalGFR_30-60” and “Sim-RenalGFR_less_30” populations available in the Simcyp Simulator were modified for use in patients with kidney dysfunction. The default kidney dysfunction populations have increased AAG concentrations, reduced albumin concentrations, reduced hepatic CYP2C8 abundances and reduced hepatic CYP3A4 abundance relative to general North European populations in the Simcyp Simulator (“Sim-NEurCaucasian”). Many studies have shown that kidney dysfunction is associated with down‐regulation of protein expression of hepatic CYP2D6, CYP2C8 and OATP1B‐mediated drug clearance, with negligible effects on CYP3A4.^11-13^ However, the interpretation is challenging due to overlapping substrate specificity with CYP2C8 and OATP1B.^13^ A recent PBPK simulation using selective CYP2C8 substrates (rosiglitazone and pioglitazone), a selective OATP1B substrate (pitavastatin) and a substrate of both proteins (repaglinide) suggested that OATP1B activity could be reduced by up to 60% in severe chronic kidney disease, with negligible effects on CYP2C8 activity.^14^ Furthermore, the N-desmethyl imatinib to imatinib area under the concentration-time curve (AUC) ratio appears unchanged in patients with mild to moderate kidney dysfunction and normal kidney function,^15^ supporting the lack of significant changes of CYP3A4 and CYP2C8 abundances in kidney dysfunction. As such, the CYP2C8 and CYP3A4 abundances in the default “Sim‐RenalGFR_30to60” and “Sim‐RenalGFR_less30” populations were changed to default values found in the general populations. CYP2C8 abundance was changed from 13.1 pmol mg^-1^ protein (“Sim‐RenalGFR_30to60”) and 11.3 pmol mg^-1^ protein (“Sim‐RenalGFR_less30”) to 24.0 pmol mg^-1^ protein in modified kidney dysfunction populations. Similarly, CYP3A4 abundance was changed from 95.2 pmol mg^-1^ protein (“Sim‐RenalGFR_30to60”) and 87.3 pmol mg^-1^ protein (“Sim‐RenalGFR_less30”) to 137.0 pmol mg^-1^ protein in modified kidney dysfunction populations.

The “Sim-NEurCaucasian” population available in the Simcyp library was used for patients of European ancestry, whereas the “Sim-Chinese”^8^ and “Sim-Japanese”^9^ populations were used for patients of Chinese and Japanese ancestry, respectively. Patients of other geographic ancestries were excluded from the simulations due to the lack of validated representative populations in the Simcyp software. Additionally, patients from the real-world observational study who had kidney or hepatic impairment, and were of Japanese or Chinese ancestry, were excluded from the simulations due to a lack of validated organ dysfunction populations in these ethnic groups in the Simcyp simulation software. The “Sim-NEurCaucasian”, “Sim-Chinese” and “Sim-Japanese” population models available in the Simcyp library incorporate known inter-ethnic physiological differences, including differences in liver volume, plasma protein composition, enzyme abundances and phenotypes, height and weight distribution, and gastrointestinal transit times. Numerous studies have demonstrated the utility of PBPK predictions in European, Chinese and Japanese virtual populations as reliable estimates of the observed PK for a number of CYP3A substrates.^8,16,17^ As previous studies have demonstrated similar abundance and metabolic capacity for CYP2C8 in European and Chinese human liver microsomes,^18,19^ we changed the mean hepatocyte CYP2C8 abundance in the “Sim-Chinese” population from 7.7 (CV 81%) to 24.0 (CV 81%) pmol mg^-1^ protein in our simulations, to mirror CYP2C8 abundance in European populations. Additionally, mean AAG abundance was modified in the “Sim-Chinese” population to 0.65 g L^-1^ (CV 15%) in males and 0.64 g L^-1^ (CV 13%) in females, mirroring the previously established concentrations in healthy Chinese populations.^16,20,21^ The imatinib PBPK model by Adiwidjaja et al^1^ was recently verified in Japanese (default) and Chinese (modified CYP2C8 and AAG abundances as detailed above) virtual populations available in the Simcyp Simulator, yielding predicted PK parameters within 1.25-fold (0.80 – 1.25) of clinically observed values.^22^

SUPPLEMENTARY TABLES

Supplementary Table 1: Drug-related parameters of the previously developed and verified physiologically based pharmacokinetic (PBPK) model for imatinib^1^ in Simcyp Simulator

| **Parameter** | **Value** |
| --- | --- |
| **Physiochemical and blood-binding properties** | |
| Molecular Weight | 493.60 |
| Log P_o:w_ | 1.99 |
| Ionisation pattern | Diprotic base |
| pKa | 8.07; 3.73 |
| B/P | 0.73 |
| fu_p_ | 0.05 |
| Plasma binding component | α_1_-acid glycoprotein |
| **Absorption phase** | |
| Model | ADAM model |
| P_eff_ (10^-4^ cm s^-1^) | 0.92 |
| fu_G_ | 1.00 |
| Q_gut_ (L h^-1^) | 6.04 |
| **Distribution phase** | |
| Prediction method | Rodgers and Rowland method |
| V_ss_ (L kg^-1^) | 1.80 |
| **Elimination phase** | |
| *Pathway 1* | CYP3A4 (NDMI formation) |
| V_max_ (pmol min^-1^ pmol CYP^-1^) | 3.00 |
| K_m_ (µmol L^-1^) | 10.54 |
| fu_inc_ | 0.96 |
| ISEF | 0.21 |
| *Pathway 2* | CYP2C8 (NDMI formation) |
| V_max_ (pmol min^-1^ pmol CYP^-1^) | 56.40 |
| K_m_ (µmol L^-1^) | 7.49 |
| fu_inc_ | 0.97 |
| *Pathway 3* | CYP3A4 (other metabolites) |
| CL_int_ (µL min^-1^ mg protein^-1^) | 33.40 |
| fu_inc_ | 1.00 |
| *Pathway 4* | CYP2C8 (other metabolites) |
| CL_int_ (µL min^-1^ mg protein^-1^) | 24.20 |
| fu_inc_ | 1.00 |
| CL_R_ (L h^-1^) | 0.50 |
| *Additional HLM* | Compensatory clearance for autoinhibition of CYP3A4 at steady-state |
| CL_int_ (µL min^-1^ mg protein^-1^) | 31.00 |
| **Drug transport-hepatobiliary transporters** | |
| *Pathway 1* | MDR1 |
| CL_int,T_ (µL min^-1^ million cells^-1^) | 1.50 |
| RAF | 1.00 |
| *Pathway 2* | BCRP |
| J_max_ (pmol min^-1^ million cells^-1^) | 89.40 |
| K_m_ (µmol L^-1^) | 4.37 |
| RAF | 0.38 |
| CL_PD_ (mL min^-1^ million hepatocytes^-1^) | 0.20 |
| **Drug interactions (for multiple dosing of imatinib)** | |
| Mechanism-based inhibition |  |
| K_inact,CYP3A_ (h^-1^) | 4.29 |
| K_I_ (µmol L^-1^) | 14.30 |
| fu_inc_ | 0.80 |

ADAM, advanced dissolution, absorption and metabolism; B/P, blood to plasma ratio; BCRP, breast cancer resistance protein; CL_int_, hepatic intrinsic clearance; CL_int,T_, transportermediated intrinsic clearance; CL_PD_, passive diffusion clearance; CL_R_, renal clearance; fu_inc_, unbound fraction during incubation; fu_G_, unbound fraction in the enterocytes; fu_p_, unbound fraction in plasma; HLM, human liver microsomes; ISEF, intersystem extrapolation factor; J_max_, maximum flux of a substrate across a drug transporter; K_I_, the concentration that provides half of K_inact_; K_inact_ ,maximum inactivation rate of CYP enzyme; K_m_, substrate concentration giving half of V_max_ or J_max_; Log P_o:w_, the partition coefficient in oil and water; MDR1, multidrug resistance protein 1 or p-glycoprotein; NDMI, N-desmethyl imatinib; P_eff_, the effective intestinal permeability; pKa, negative logarithm of acid dissociation constant; Q_Gut_, the gut blood flow rate; RAF, relative activity factor; V_max_,maximumrate of reaction; V_ss_, volume of distribution at steady-state based on total tissue volumes.

Supplementary Table 2: Comparison of predicted pharmacokinetic parameter values of imatinib using the Simcyp Simulator version 18, with those predicted using version 17 (Adiwidjaja et al., 2020)^1^ and clinically reported values

| **Population** | **Age range (years)** | **Dose Regimen** | **PK parameter** | **Clinically observed value** | **PBPK model prediction (using Simcyp version 17)**^1^ | **PBPK model prediction (using Simcyp version 18)** | | **Prediction fold difference to clinically observed value** | |
| --- | --- | --- | --- | --- | --- | --- | --- | --- | --- |
|  |  |  |  |  |  | **Default population in version 18** | **Modified population in version 18^(c)^** | **Default population in version 18** | **Modified population in version 18^(c)^** |
| Patients with GIST (n=34; 6 female)^23^ | 28 – 84 | 400 mg/day at steady-state | CL/F (L h^-1^) | 10.9^(a)^ | 10.7 | 11.41 | 10.87 | 1.05 | 1 |
|  |  |  | CV of CL/F (%) | 19^(b)^ | 54 | 61 | 58 | - | - |
| Patients with PAH (n=103; 83 female)^24^ | 18 – 77 | 400 mg/day at steady-state | CL/F (L h^-1^) | 10.8^(a)^ | 9.8 | 11.04 | 10.02 | 1.02 | 0.93 |
|  |  |  | CV of CL/F (%) | 43^(b)^ | 53 | 57 | 53 | - | - |
| Patients with GIST (n=50; 21 female)^25^ | 39 – 82 | 74% received 400 mg/day at steady-state, with 26% receiving 800 mg/day | CL/F (L h^-1^) | 9.1^(a)^ | 9.6 | 10.40 | 9.8 | 1.14 | 1.08 |
|  |  |  | CV of CL/F (%) | 50^(b)^ | 52 | 59 | 56 | - | - |

PBPK model predictions are reported as geometric mean values.

(a) Typical population value.

(b) Based on ω (standard deviation of eta, interindividual variability) of CL/F.

(c) Mean abundance of α_1_-acid glycoprotein in v18 was modified to the following values used in v17; males: 0.811 (CV 15%) and females: 0.791 (CV 13%).

CL/F, apparent clearance; CV, coefficient of variation; GIST, gastrointestinal stromal tumours; PAH, pulmonary arterial hypertension; PBPK, physiologically based pharmacokinetic; PK, pharmacokinetics.

Supplementary Table 3: PBPK model input variables and predicted imatinib PK parameters of patients followed in the real-world retrospective cohort study,^2^ based on achievement of EMR at 3 months

| **EMR achieved** | **Imatinib dose** | **Geographic ancestry** | **Kidney/ hepatic dysfunction** | **Simcyp population** | **n subjects per trial**  **(females)** | **Age range, years** | **Weight range, kg** | **Concomitant medicine (dose)** | **PBPK predicted imatinib PK parameters** | | | |
| --- | --- | --- | --- | --- | --- | --- | --- | --- | --- | --- | --- | --- |
|  |  |  |  |  |  |  |  |  | **AUC_0-24,ss_**  **µg h mL^-1^ (CV%)** | **C_ss,min_**  **µg mL^-1^ (CV%)** | **C_ss,max_**  **µg mL^-1^ (CV%)** | **CL/F**  **L h^-1^ (CV%)** |
| No | 300 mg daily | European | GFR 30-60 mL/min/1.73m² | modified Sim-RenalGFR_30-60^(a)^ | 2 (1) | 74 – 85 | 72 – 98 | - | 40.28 (0.44) | 0.98 (0.59) | 2.47 (0.35) | 7.45 (0.35) |
| No | 400 mg daily | European | No | Sim-NEurCaucasian | 1 (0) | 39 | 118 | - | 23.87 (0.32) | 0.50 (0.52) | 1.56 (0.21) | 16.76 (0.31) |
| No | 400 mg daily | European | GFR 30-60 mL/min/1.73m² | modified Sim-RenalGFR_30-60^(a)^ | 1 (1) | 72 | 63 | - | 45.92 (0.42) | 0.85 (0.64) | 3.21 (0.30) | 8.71 (0.53) |
| No | 400 mg daily | Chinese | No | modified Sim-Chinese^(b)^ | 1 (0) | 22 | 65 | - | 30.13 (0.34) | 0.51 (0.52) | 2.22 (0.23) | 13.28 (0.55) |
| No | 500 mg daily | European | No | Sim-NEurCaucasian | 1 (0) | 45 | 120 | - | 31.78 (0.33) | 0.69 (0.52) | 2.04 (0.22) | 15.73 (0.31) |
| No | 600 mg daily | European | No | Sim-NEurCaucasian | 3 (0) | 32 – 75 | 70 – 90 | - | 62.61 (0.72) | 1.32 (0.99) | 4.19 (0.55) | 9.58 (0.74) |
| No | 400 mg daily | European | No | Sim-NEurCaucasian | 1 (0) | 66.4 | 86 | verapamil (60 mg every 8 hours) | 47.96 (0.30) | 1.15 (0.60) | 2.98 (0.27) | 8.34 (0.36) |
| Yes | 400 mg daily | European | No | Sim-NEurCaucasian | 8 (5) | 26 – 79 | 51 – 128 | - | 41.68 (0.54) | 0.76 (0.83) | 2.99 (0.37) | 9.60 (0.62) |
| Yes | 400 mg daily | European | GFR 30-60 mL/min/1.73m² | modified Sim-RenalGFR_30-60^(a)^ | 3 (1) | 61 – 87 | 74 – 79 | - | 62.03 (0.41) | 1.54 (0.57) | 3.81 (0.30) | 6.45 (0.52) |
| Yes | 400 mg daily | European | GFR < 30 mL/min/1.73m² | modified Sim-RenalGFR_less_30^(a)^ | 1 (0) | 70 | 100 | - | 50.06 (0.46) | 1.27 (0.63) | 2.91 (0.35) | 7.99 (0.45) |
| Yes | 400 mg daily | European | Mild hepatic impairment | Sim-CirrhosisCP-A | 1 (1) | 78 | 67 | - | 61.5 (0.30) | 1.42 (0.47) | 3.82 (0.20) | 6.5 (0.29) |
| Yes | 400 mg daily | Chinese | No | modified Sim-Chinese^(b)^ | 1 (1) | 66 | 77 | - | 40.73 (0.36) | 0.76 (0.52) | 2.87 (0.25) | 9.82 (0.57) |
| Yes | 600 mg daily | European | No | Sim-NEurCaucasian | 11 (4) | 36 – 74 | 68 – 104 | - | 54.40 (0.56) | 1.05 (0.86) | 3.79 (0.39) | 11.03 (0.53) |
| Yes | 600 mg daily | European | GFR 30-60 mL/min/1.73m² | modified Sim-RenalGFR_30-60^(a)^ | 1 (0) | 84 | 109 | - | 81.78 (0.38) | 1.60 (0.53) | 3.84 (0.30) | 9.32 (0.50) |
| Yes | 600 mg daily | Chinese | No | modified Sim-Chinese^(b)^ | 2 (0) | 25 – 40 | 77 | - | 42.09 (0.42) | 0.74 (0.65) | 3.10 (0.29) | 14.25 (0.61) |
| Yes | 600 mg daily | Japanese | No | Sim-Japanese | 1 (0) | 23 | 74 | - | 46.83 (0.35) | 0.88 (0.52) | 3.22 (0.24) | 12.81 (0.53) |
| Yes | 400 mg morning and 200 mg night | European | No | Sim-NEurCaucasian | 1 (0) | 34 | 116 | - | 42.06 (0.33) | 1.17 (0.45) | 2.35 (0.25) | 10.45 (0.33) |
| Yes | 400 mg twice daily | European | No | Sim-NEurCaucasian | 2 (0) | 56 – 61 | 74 – 103 | - | 72.16 (0.56) | 2.23 (0.65) | 3.76 (0.49) | 11.07 (0.52) |
| Yes | 600 mg daily | European | GFR 30-60 mL/min/1.73m² | modified Sim-RenalGFR_30-60^(a)^ | 1 (1) | 62 | 74 | clopidogrel (75 mg daily) | 76.57 (0.46) | 1.56 (0.68) | 4.96 (0.34) | 7.84 (0.49) |
| Yes | 400 mg daily | European | No | Sim-NEurCaucasian | 1 (0) | 69 | 75 | clopidogrel (75 mg daily) | 69.92 (0.31) | 1.94 (0.43) | 3.95 (0.23) | 5.72 (0.26) |
| Yes | 400 mg daily | European | No | Sim-NEurCaucasian | 1 (0) | 56 | 96 | diltiazem (45 mg every 6 hours) | 31.09 (0.35) | 0.64 (0.51) | 2.06 (0.25) | 12.87 (0.51) |

The Simcyp population used, number of virtual subjects, number of females, age range, weight range, and any drug-drug interactions in each PBPK simulation is detailed. Imatinib dosing regimen over 14 days is also specified for each simulation, in addition to the clinical outcome category. A total of 10 trials for each simulation were carried out. All pharmacokinetic parameters are reported as geometric mean values and percentage of coefficient of variation (CV%) of PBPK model predictions at day 14.

(a) CYP2C8 and CYP3A4 abundances in the default “Sim‐RenalGFR_30to60” and “Sim‐RenalGFR_less30” populations were changed to default values found in healthy populations

(b) mean AAG abundances and hepatic CYP2C8 abundances in the default "Sim-Chinese" population were modified.

AUC_0-24,ss_, area under the plasma concentration-time curve from time zero to 24 hours after a dose at steady-state; C_ss,max_, maximum (peak) plasma concentration at steady-state; C_ss,min_ , minimum (trough) plasma concentration at steady-state; CL/F, apparent clearance; EMR, early molecular response at 3 months; GFR, glomerular filtration rate; PBPK, physiologically based pharmacokinetic; PK, pharmacokinetic.

Supplementary Table 4: PBPK model input variables and predicted imatinib PK parameters of patients followed in the real-world retrospective cohort study,^2^ based on occurrence of imatinib-related grade ≥3 ADRs

| **Occurrence of a grade ≥3 ADR** | **Imatinib dose** | **Geographic ancestry** | **Kidney/ hepatic dysfunction** | **Simcyp population** | **n subjects per trial**  **(females)** | **Age range, years** | **Weight range, kg** | **Concomitant medicine (dose)** | **PBPK predicted imatinib PK parameters** | | | |
| --- | --- | --- | --- | --- | --- | --- | --- | --- | --- | --- | --- | --- |
|  |  |  |  |  |  |  |  |  | **AUC_0-24,ss_**  **µg h mL^-1^ (CV%)** | **C_ss,min_**  **µg mL^-1^ (CV%)** | **C_ss,max_**  **µg mL^-1^ (CV%)** | **CL/F**  **L h^-1^ (CV%)** |
| No | 800 mg daily | European | No | Sim-NEurCaucasian | 1 (1) | 57 | 50 | - | 115.66 (0.33) | 2.24 (0.57) | 8.22 (0.21) | 6.92 (0.29) |
| No | 400 mg twice daily | European | No | Sim-NEurCaucasian | 4 (0) | 34 – 65 | 74 – 120 | - | 59.32 (0.48) | 1.80 (0.59) | 3.11 (0.41) | 13.47 (0.44) |
| No | 600 mg daily | European | No | Sim-NEurCaucasian | 1 (0) | 53 | 128 | - | 35.58 (0.39) | 0.70 (0.59) | 2.42 (0.28) | 16.86 (0.50) |
| No | 400 mg daily | European | No | Sim-NEurCaucasian | 9 (4) | 36 – 79 | 51 – 118 | - | 39.73 (0.45) | 0.77 (0.67) | 2.76 (0.35) | 10.07 (0.69) |
| No | 400 mg daily | European | GFR 30-60 mL/min/1.73m² | modified Sim-RenalGFR_30-60^(a)^ | 1 (0) | 62 | 74 | - | 34.25 (0.39) | 0.67 (0.59) | 2.23 (0.28) | 11.68 (0.50) |
| No | 300 mg daily | European | GFR 30-60 mL/min/1.73m² | modified Sim-RenalGFR_30-60^(a)^ | 1 (0) | 75 | 98 | - | 25.82 (0.39) | 0.58 (0.57) | 1.62 (0.29) | 11.62 (0.49) |
| No | 800 mg daily | Chinese | No | modified Sim-Chinese (b) | 1 (0) | 26 | 77 | - | 68.71 (0.36) | 1.39 (0.59) | 4.65 (0.24) | 11.64 (0.31) |
| No | 400 mg daily | European | Mild hepatic impairment | Sim-CirrhosisCP-A | 1 (1) | 79 | 67 | clopidogrel (75 mg daily) for 14 days | 78.69 (0.29) | 2.04 (0.42) | 4.55 (0.21) | 5.08 (0.26) |
| No | 300 mg daily | European | GFR 30-60 mL/min/1.73m² | modified Sim-RenalGFR_30-60^(a)^ | 1 (1) | 71 | 70 | clopidogrel (75 mg daily) for 14 days | 52.20 (0.48) | 1.25 (0.67) | 3.14 (0.36) | 5.75 (0.42) |
| No | 400 mg daily | European | GFR 30-60 mL/min/1.73m² | modified Sim-RenalGFR_30-60^(a)^ | 1 (0) | 56 | 96 | diltiazem (45 mg every 6 hours) for 14 days | 38.87 (0.32) | 0.91 (0.46) | 2.38 (0.24) | 10.29 (0.42) |
| Yes | 800 mg daily | European | No | Sim-NEurCaucasian | 1 (0) | 58 | 89 | - | 82.85 (0.34) | 1.95 (0.52) | 5.16 (0.23) | 9.66 (0.30) |
| Yes | 400 mg morning and 200 mg night | European | No | Sim-NEurCaucasian | 1 (0) | 33.7 | 116 | - | 41.90 (0.33) | 1.16 (90.45) | 2.35 (0.25) | 10.47 (0.33) |
| Yes | 600 mg daily | European | No | Sim-NEurCaucasian | 16 (6) | 35 – 76 | 54 – 104 | - | 61.96 (0.51) | 1.22 (0.77) | 4.28 (0.36) | 9.68 (0.57) |
| Yes | 600 mg daily | European | GFR 30-60 mL/min/1.73m² | modified Sim-RenalGFR_30-60^(a)^ | 1 (1) | 72 – 84 | 63 – 109 | - | 86.46 (0.47) | 2.15 (0.60) | 5.30 (0.38) | 6.94 (0.58) |
| Yes | 300 mg twice daily | European | No | Sim-NEurCaucasian | 1 (0) | 61 | 74 | - | 76.17 (0.33) | 2.47 (0.41) | 3.81 (0.28) | 7.86 (0.30) |
| Yes | 400 mg daily | European | No | Sim-NEurCaucasian | 9 (4) | 26 – 86 | 60 – 100 | - | 41.06 (0.54) | 0.79 (0.87) | 2.88 (0.37) | 9.74 (0.46) |
| Yes | 400 mg daily | European | GFR 30-60 mL/min/1.73m² | modified Sim-RenalGFR_30-60^(a)^ | 4 (3) | 65 – 87 | 73 – 79 | - | 57.88 (0.45) | 1.34 (0.64) | 3.68 (0.32) | 6.91 (0.62) |
| Yes | 400 mg daily | European | GFR < 30 mL/min/1.73m² | modified Sim-RenalGFR_less_30^(a)^ | 1 (0) | 70 | 100 | - | 49.37 (0.46) | 1.25 (0.63) | 2.88 (0.35) | 8.10 (0.45) |
| Yes | 400 mg daily | European | Mild hepatic impairment | Sim-CirrhosisCP-A | 1 (0) | 78 | 80 | - | 46.73 (0.43) | 1.08 (0.62) | 2.85 (0.32) | 8.56 (0.44) |
| Yes | 300 mg daily | European | GFR 30-60 mL/min/1.73m² | modified Sim-RenalGFR_30-60^(a)^ | 1 (0) | 73 | 55 | - | 37.14 (0.43) | 0.69 (0.65) | 2.63 (0.30) | 8.08 (0.52) |
| Yes | 600 mg daily | Chinese | No | modified Sim-Chinese^(b)^ | 4 (1) | 27 – 66 | 65 – 77 | - | 63.81 (0.43) | 1.34 (0.61) | 4.29 (0.30) | 9.40 (0.42) |
| Yes | 400 mg daily | Chinese | No | modified Sim-Chinese^(b)^ | 1 (1) | 57 | 73 | - | 44.94 (0.36) | 0.90 (0.59) | 3.06 (0.24) | 8.90 (0.31) |
| Yes | 400 mg twice daily | Japanese | No | Sim-Japanese | 1 (0) | 27 | 74 | - | 63.80 (0.34) | 1.91 (0.42) | 3.33 (0.29) | 12.52 (0.52) |
| Yes | 400 mg daily | European | No | Sim-NEurCaucasian | 1 (0) | 69 | 75 | clopidogrel (75 mg daily) for 14 days | 70.34 (0.31) | 1.95 (0.43) | 3.97 (0.23) | 5.69 (0.26) |
| Yes | 400 mg twice daily | European | No | Sim-NEurCaucasian | 1 (0) | 66 | 74 | St John’s Wort (45 mg daily) for 14 days | 102.16 (0.18) | 3.33 (0.23) | 5.11 (0.15) | 7.82 (0.18) |
| Yes | 500 mg daily | European | No | Sim-NEurCaucasian | 2 (0) | 71 | 91 | trimethoprim (300 mg daily) for 7 days | 59.41 (0.30) | 1.42 (0.40) | 3.61 (0.23) | 8.42 (0.45) |
| Yes | 400 mg daily | European | No | Sim-NEurCaucasian | 1 (0) | 69 | 86 | verapamil (80 mg every 8 hours) for 14 days | 52.07 (0.40) | 1.29 (0.59) | 3.18 (0.27) | 7.68 (0.35) |

The Simcyp population used, number of virtual subjects, number of females, age range, weight range, and any drug-drug interactions in each PBPK simulation is detailed. Imatinib dosing regimen over 14 days is also specified for each simulation, in addition to the clinical outcome category. A total of 10 trials for each simulation were carried out. All pharmacokinetic parameters are reported as geometric mean values and percentage of coefficient of variation (CV%) of PBPK model predictions at day 14.

(a) CYP2C8 and CYP3A4 abundances in the default “Sim‐RenalGFR_30to60” and “Sim‐RenalGFR_less30” populations were changed to default values found in healthy populations

(b) mean AAG abundances and hepatic CYP2C8 abundances in the default "Sim-Chinese" population were modified.

ADR, adverse drug reaction; AUC_0-24,ss_, area under the plasma concentration-time curve from time zero to 24 hours after a dose at steady-state;; C_ss,max_, maximum (peak) plasma concentration at steady-state; C_ss,min_ , minimum (trough) plasma concentration at steady-state; CL/F, apparent clearance; GFR, glomerular filtration rate; PBPK, physiologically based pharmacokinetic; PK, pharmacokinetic.

Supplementary Table 5: Summary of PBPK model predicted imatinib exposure when co-administered with specific CYP modulators at steady-state

| **CYP modulator and dosing regimen** | **PK parameter** | **Geometric mean PBPK model prediction using Simcyp version 18 (CV%)** | | **Predicted PK parameter ratio (90% CI)** |
| --- | --- | --- | --- | --- |
|  |  | **Imatinib alone** | **Imatinib plus CYP modulator** |  |
| Diltiazem 45 mg every 6 hours, for 14 days (from day 1) | AUC_0-24,ss_ (µg h mL^-1^) | 38.47 (0.66) | 40.08 (0.65) | 1.04 (1.04—1.05) |
|  | C_ss,max_ (µg mL^-1^) | 2.71 (0.48) | 2.79 (0.48) | 1.03 (1.03—1.03) |
|  | C_ss,min_ (µg mL^-1^) | 0.73 (0.99) | 0.78 (0.96) | 1.07 (1.06—1.08) |
|  | CL/F (L h^-1^) | 10.40 (0.59) | 9.98 (0.58) | 0.96 (0.96—0.96) |
| Verapamil 60 mg every 8 hours, for 14 days (from day 1) | AUC_0-24,ss_ (µg h mL^-1^) | 38.26 (0.65) | 40.57 (0.63) | 1.06 (1.06—1.07) |
|  | C_ss,max_ (µg mL^-1^) | 2.71 (0.47) | 2.83 (0.46) | 1.04 (1.04—1.05) |
|  | C_ss,min_ (µg mL^-1^) | 0.72 (0.97) | 0.79 (0.94) | 1.10 (1.09—1.11) |
|  | CL/F (L h^-1^) | 10.46 (0.59) | 9.86 (0.59) | 0.94 (0.94—0.95) |
| St John's Wort (hyperforin) 45 mg daily for 14 days (from day 1) | AUC_0-24,ss_ (µg h mL^-1^) | 38 (0.58) | 36.19 (0.59) | 0.95 (0.95—0.96) |
|  | C_ss,max_ (µg mL^-1^) | 2.69 (0.43) | 2.59 (0.43) | 0.96 (0.96—0.97) |
|  | C_ss,min_ (µg mL^-1^) | 0.72 (0.88) | 0.67 (0.89) | 0.93 (0.92—0.94) |
|  | CL/F (L h^-1^) | 10.53 (0.57) | 11.05 (0.57) | 1.05 (1.05—1.05) |
| Clopidogrel 75 mg daily for 14 days (from day 1) | AUC_0-24,ss_ (µg h mL^-1^) | 37.67 (0.63) | 49.65 (0.59) | 1.32 (1.28—1.36) |
|  | C_ss,max_ (µg mL^-1^) | 2.67 (0.46) | 3.20 (0.46) | 1.20 (1.17—1.22) |
|  | C_ss,min_ (µg mL^-1^) | 0.71 (0.94) | 1.09 (0.83) | 1.54 (1.46—1.61) |
|  | CL/F (L h^-1^) | 10.62 (0.57) | 8.06 (0.55) | 0.76 (0.74—0.78) |
| Trimethoprim 300 mg daily for 7 days (from day 8) | AUC_0-24,ss_ (µg h mL^-1^) | 37.68 (0.68) | 46.19 (0.62) | 1.23 (1.20—1.25) |
|  | C_ss,max_ (µg mL^-1^) | 2.68 (0.49) | 3.08 (0.46) | 1.15 (1.14—1.16) |
|  | C_ss,min_ (µg mL^-1^) | 0.71 (1.04) | 0.95 (0.92) | 1.34 (1.31—1.38) |
|  | CL/F (L h^-1^) | 10.61 (0.59) | 8.66 (0.55) | 0.82 (0.80—0.83) |

Simulations were performed using a North European Caucasian population (10 subjects, 4 females, age range 20 to 91 years) with a multiple-dosing regimen of imatinib 400 mg daily for 14 days. The dosing regimen of the modulators in the PBPK simulations are detailed. A total of 10 trials for each simulation were carried out.

All pharmacokinetic parameters are reported as geometric mean values and percentage of coefficient of variation (CV%). The extent of interactions was evaluated based on predicted pharmacokinetic parameter ratios (ratio in the presence and absence of CYP modulators) and their 90% confidence intervals. A ratio of 1 indicates absence of drug interactions with imatinib.

AUC_0-24,ss_, area under the plasma concentration-time curve from time zero to 24 hours after a dose at steady-state; C_ss,max_ ,maximum (peak) plasma concentration at steady-state; C_ss,min_ , minimum (trough) plasma concentration at steady-state; CL/F, apparent clearance; CYP, cytochrome P450; PBPK, physiologically based pharmacokinetic; PK, pharmacokinetic.

SUPPLEMENTARY FIGURES

Supplementary Figure 1: Sensitivity analyses results showing the effect of changes in input parameters on the imatinib PK profiles in virtual patients. Sensitivity analyses were conducted for: **A)** age (years), **B)** sex, **C)** actual body weight (kg), **D)** dose (mg/day), **E)** serum AAG concentrations (g L^-1^), **F)** intestinal CYP3A4 abundance (pmol mg^-1^protein), **G)** hepatic CYP3A4 abundance (pmol mg^-1^ protein), **H)** hepatic CYP2C8 abundance (pmol mg^-1^ protein), **I)** hepatobiliary P-gp abundance (pmol 10^6^ hepatocytes^-1^), **J)** hepatobiliary BCRP abundance (pmol 10^6^ hepatocytes^-1^), **K)** hepatic impairment (defined using Cirrhosis scores), **L)** kidney dysfunction (using default GFR populations), **M)** kidney dysfunction (using GFR populations with modified CYP abundances adjusted to that of the general population).

(A)

(B)

Simulation results are depicted as geometric mean predicted plasma concentrations of imatinib (ng mL^-1^) with variations in input parameters represented by different coloured lines. Simulations were conducted using imatinib 400 mg daily (except when analysing variations in dose), for 14 days (to achieve steady-state).

AAG, α_1_-acid glycoprotein; BCRP, breast cancer resistant protein; CYP, cytochrome P450; GFR, glomerular filtration rate; P-gp, p-glycoprotein; PK, pharmacokinetic.

Supp Figure 1 cont.

(C)

(D)

g L^-1^

g L^-1^

g L^-1^

g L^-1^

g L^-1^

(F)

(E)

Supp Figure 1 cont.

(G)

(H)

(I)

(J)

(K)

(L)

(M)

Supp Figure 1 cont.

Commentary on sensitivity analysis

Supplementary Figure 1 presents results of the sensitivity analyses.

The simulated plasma concentration-time profile of imatinib was sensitive to variation in subject age, in which a change in age from 20 to 55 years decreased apparent clearance (CL/F) from 14.47 to 11.24 L h^-1^ (*P* < 0.05), increased the steady-state area under the plasma concentration-time curve during 24 hours after the dose (AUC _0-24,ss_) from 27.63 to 35.58 µg h mL^-1^ (*P* < 0.05), increased steady-state maximum concentration (C_ss,max_) from 1.95 to 2.43 µg mL^-1^ (*P* < 0.05), and increased steady-state trough plasma concentration (C_ss,min_) from 0.50 to 0.73 µg mL^-1^ (*P* < 0.05). A change in age from 55 to 90 years decreased CL/F from 11.24 to 5.54 L h^-1^ (*P* < 0.05), increased the AUC _0-24,ss_ from 35.58 to 72.14 µg h mL^-1^ (*P* < 0.05), increased C_ss,max_ from 2.43 to 4.27 µg mL^-1^ (*P* < 0.05), and increased C_ss,min_ from 0.73 to 1.92 µg mL^-1^ (*P* < 0.05).

Furthermore, imatinib plasma concentration-time profiles were sensitive to variation in ABW, whereby a change in ABW from 45 to 125 kg increased CL/F from 8.05 to 14.32 L h^-1^ (*P* < 0.05), decreased AUC _0-24,ss_ from 49.70 to 27.94 µg h mL^-1^ (*P* < 0.05), decreased C_ss,max_ from 3.60 to 1.89 µg mL^-1^ (*P* < 0.05), and decreased C_ss,min_ from 0.96 to 0.58 µg mL^-1^ (*P* < 0.05).

The peak plasma concentration of imatinib was sensitive to sex, with females showing an 18.2% higher C_ss,max_  than males (*P <* 0.05). However, other parameters were not significantly different, with only a 6% lower CL/F in females compared to males (P = 0.53), 7% higher AUC _0-24,ss_ (*P* = 0.55) and 15% lower C_ss,min_ (*P* = 0.43). These changes in imatinib exposure are unlikely to be of clinical significance.

The simulated plasma concentration-time profiles of imatinib were sensitive to variation in imatinib daily dose, whereby a dosage range from 100 to 800 mg daily increased AUC_0-24,ss_ from 7.07 to 71.48 µg h mL^-1^ (*P* < 0.05), increased C_ss,max_ from 0.51 to 4.81 µg mL^-1^ (*P* < 0.05), and increased C_ss,min_ from 0.13 to 1.54 µg mL^-1^ (*P* < 0.05).

Additionally, the predicted plasma concentration-time profiles of imatinib were sensitive to variation in AAG concentrations. As AAG concentration increased from 0.3 to 3.2 mg/mL, CL/F decreased from 25.20 to 4.59 L h^-1^ (*P* < 0.05), total plasma imatinib AUC_0-24,ss_ increased from 13.99 to 117.19 µg h mL^-1^ (*P* < 0.05), C_ss,max_ increased from 1.29 to 5.91 µg mL^-1^ (*P* < 0.05), and C_ss,min_ increased from 0.15 to 3.89 µg mL^-1^ (*P* < 0.05).

The predicted plasma concentration-time profiles of imatinib were sensitive to variation in hepatic CYP2C8 and CYP3A4 enzyme abundances, but to different magnitudes. A hepatic CYP2C8 abundance range of 0 to 85.00 pmol mg^-1^ protein increased CL/F from 10.52 to 20.79 L h^-1^ (*P* < 0.05), decreased AUC_0-24,ss_ from 58.79 to 17.82 µg h mL^-1^ (*P* < 0.05), decreased C_ss,max_ from 3.40 to 1.55 µg mL^-1^ (*P* < 0.05), and decreased C_ss,min_ from 1.6 to 0.24 µg mL^-1^. A hepatic CYP3A4 enzyme abundance range of 0 to 600.00 pmol mg^-1^ protein increased CL/F from 9.34 to 24.69 L h^-1^ (*P* < 0.05), decreased AUC_0-24,ss_ from 38.96 to 19.70 µg h mL^-1^ (*P* < 0.05), decreased C_ss,max_ from 2.55 to 1.63 µg mL^-1^ (*P* < 0.05), and decreased C_ss,min_ from 0.88 to 0.28 µg mL^-1^ (*P* < 0.05). Conversely, simulated imatinib plasma concentration-time profiles were not sensitive to variation in intestinal CYP3A4 enzyme abundance, or changes in hepatobiliary P-gp and BCRP transporter abundances .

Simulated imatinib plasma concentration-time profiles were sensitive to varying degrees of hepatic impairment (measured as liver cirrhosis). Adults with Child-Pugh B cirrhosis had a 26% reduction in imatinib CL/F compared to healthy (“Sim-NEurCaucasian”) adults (*P* < 0.05), whilst adults with Child-Pugh C cirrhosis had a 40% reduction in imatinib CL/F compared to healthy adults (*P* < 0.05). Accordingly, Child-Pugh B cirrhosis resulted in a 36% higher AUC_0-24,ss_ (*P* < 0.05), 18% higher C_ss,max_ (*P* < 0.05) and 56% higher C_ss,min_ (*P* < 0.05) compared to healthy adults. Child-Pugh C cirrhosis was associated with a 66% increase in AUC_0-24,ss_ (*P* < 0.05), 42% increase in C_ss,max_ (*P* < 0.05) and 100% increase in C_ss,min_ (*P* < 0.05) relative to healthy adults. There was no significant difference in plasma-concentration time profile between Child-Pugh A cirrhosis and healthy adults.

Imatinib exposure was sensitive to variations in glomerular filtration rate (GFR) when simulated using default kidney dysfunction populations. Simulations demonstrated a 35% reduction in imatinib CL/F if GFR was between 30 to 60 mL/min/1.73 m² (*P* < 0.05) and a 62% reduction in imatinib CL/F if GFR was less than 30 mL/min/1.73 m² (*P* < 0.05) relative to the “Sim-NEurCaucasian” population (normal kidney function). Accordingly, GFR between 30 to 60 mL/min/1.73 m² was associated with a 53% higher AUC_0-24,ss_ (*P* < 0.05), 36% higher C_ss,max_ (*P* < 0.05) and 85% higher C_ss,min_ (*P* < 0.05) compared to normal kidney function. GFR less than 30 mL/min/1.73 m² was associated with a 2.6-fold increase in AUC_0-24,ss_ (*P* < 0.05), 2.2-fold increase in C_ss,max_ (*P* < 0.05) and a 3.4-fold increase in C_ss,min_ (*P* < 0.05) relative to normal kidney function.

Simulated imatinib systemic exposure remained sensitive to kidney dysfunction in the modified GFR populations (CYP enzyme abundance changed to default values in general populations), however to a lesser extent. In the modified simulation, a GFR between 30 to 60 mL/min/1.73 m² was associated with a 20% reduction in imatinib CL/F (*P* < 0.05), whilst a GFR less than 30 mL/min/1.73 m² was associated with a 43% reduction in imatinib CL/F (*P* < 0.05). Accordingly, a GFR between 30 to 60 mL/min/1.73 m² was associated with a 25% higher AUC_0-24,ss_ (*P* < 0.05), an 18% higher C_ss,max_ (*P* < 0.05), and a 41% higher C_ss,min_ (*P* < 0.05) compared to adults with normal kidney function. GFR less than 30 mL/min/1.73 m² was associated with a 75% increase in AUC_0-24,ss_ (*P* < 0.05), a 48% increase in C_ss,max_ (*P* < 0.05), and a 2.3-fold increase in C_ss,min_ (*P* < 0.05) relative to adults with normal kidney function.

REFERENCES

1. Adiwidjaja J, Boddy AV, McLachlan AJ. Implementation of a physiologically based pharmacokinetic modeling approach to guide optimal dosing regimens for imatinib and potential drug interactions in paediatrics. *Front Pharmacol*. 2020;10:1672. doi:10.3389/fphar.2019.01672

2. Adattini JA, Gross AS, Wong Doo N, McLachlan AJ. Real-world efficacy and safety outcomes of imatinib treatment in patients with chronic myeloid leukaemia: an Australian experience. Submitted, *under review*. 2021

3. Fraeyman NF, Dello CD, Belpaire FM. Alpha 1-acid glycoprotein concentration and molecular heterogeneity: relationship to oxprenolol binding in serum from healthy volunteers and patients with lung carcinoma or cirrhosis. *Br J Clin Pharmacol*. 1988;25(6):733-40. doi:10.1111/j.1365-2125.1988.tb05260.x

4. Al-Dewik NI, Jewell AP, Yassin MA, Morsi HM. Studying the impact of presence of alpha acid glycoprotein and protein glycoprotein in chronic myeloid leukemia patients treated with imatinib mesylate in the State of Qatar. *Biomark Cancer*. 2015;7:63-7. doi:10.4137/bic.s31427

5. Jorgensen HG, Elliott MA, Allan EK, Carr CE, Holyoake TL, Smith KD. Alpha1-acid glycoprotein expressed in the plasma of chronic myeloid leukemia patients does not mediate significant in vitro resistance to STI571. *Blood*. 2002;99(2):713-5.

6. Gandia P, Arellano C, Lafont T, Huguet F, Malard L, Chatelut E. Should therapeutic drug monitoring of the unbound fraction of imatinib and its main active metabolite N-desmethyl-imatinib be developed? *Cancer Chemother Pharmacol*. 2013;71(2):531-6. doi:10.1007/s00280-012-2035-3

7. Smith P, Bullock JM, Booker BM, Haas CE, Berenson CS, Jusko WJ. The influence of St. John's wort on the pharmacokinetics and protein binding of imatinib mesylate. *Pharmacotherapy*. 2004;24(11):1508-14. doi:10.1592/phco.24.16.1508.50958

8. Wang HY, Chen X, Jiang J, Shi J, Hu P. Evaluating a physiologically based pharmacokinetic model for predicting the pharmacokinetics of midazolam in Chinese after oral administration. *Acta Pharmacol Sin*. 2016;37(2):276-84. doi:10.1038/aps.2015.122

9. Matsumoto Y, Cabalu T, Sandhu P, et al. Application of physiologically based pharmacokinetic modeling to predict pharmacokinetics in healthy Japanese subjects. *Clin Pharmacol Ther*. 2019;105(4):1018-30. doi:10.1002/cpt.1240

10. Johnson TN, Boussery K, Rowland-Yeo K, Tucker GT, Rostami-Hodjegan A. A semi-mechanistic model to predict the effects of liver cirrhosis on drug clearance. *Clin Pharmacokinet*. 2010;49(3):189-206. doi:10.2165/11318160-000000000-00000

11. Pichette V, Leblond FA. Drug metabolism in chronic renal failure. *Curr Drug Metab*. 2003;4(2):91-103. doi:10.2174/1389200033489532

12. Rowland-Yeo K, Aarabi M, Jamei M, Rostami-Hodjegan A. Modeling and predicting drug pharmacokinetics in patients with renal impairment. *Expert Rev Clin Pharmacol*. 2011;4(2):261-74. doi:10.1586/ecp.10.143

13. Tan ML, Yoshida K, Zhao P, et al. Effect of chronic kidney disease on nonrenal elimination pathways: a systematic assessment of CYP1A2, CYP2C8, CYP2C9, CYP2C19, and OATP. *Clin Pharmacol Ther*. 2018;103(5):854-67. doi:10.1002/cpt.807

14. Tan ML, Zhao P, Zhang L, et al. Use of physiologically based pharmacokinetic modeling to evaluate the effect of chronic kidney disease on the disposition of hepatic CYP2C8 and OATP1B drug substrates. *Clin Pharmacol Ther*. 2019;105(3):719-29. doi:10.1002/cpt.1205

15. Gibbons J, Egorin MJ, Ramanathan RK, et al. Phase I and pharmacokinetic study of imatinib mesylate in patients with advanced malignancies and varying degrees of renal dysfunction: a study by the National Cancer Institute Organ Dysfunction Working Group. *J Clin Oncol*. 2008;26(4):570-6. doi:10.1200/jco.2007.13.3819

16. Barter ZE, Tucker GT, Rowland-Yeo K. Differences in cytochrome p450-mediated pharmacokinetics between chinese and caucasian populations predicted by mechanistic physiologically based pharmacokinetic modelling. *Clin Pharmacokinet*. 2013;52(12):1085-100. doi:10.1007/s40262-013-0089-y

17. Matsumoto Y, Cabalu T, Sandhu P, et al. Application of physiologically based pharmacokinetic modeling to predict pharmacokinetics in healthy Japanese subjects. *Clin Pharmacol Ther*. Apr 2019;105(4):1018-1030. doi:10.1002/cpt.1240

18. Yang J, He MM, Niu W, et al. Metabolic capabilities of cytochrome P450 enzymes in Chinese liver microsomes compared with those in Caucasian liver microsomes. *Br J Clin Pharmacol*. 2012;73(2):268-84. doi:10.1111/j.1365-2125.2011.04076.x

19. Zhang HF, Wang HH, Gao N, et al. Physiological content and intrinsic activities of 10 cytochrome P450 isoforms in human normal liver microsomes. *J Pharmacol Exp Ther*. 2016;358(1):83-93. doi:10.1124/jpet.116.233635

20. Feely J, Grimm T. A comparison of drug protein binding and alpha 1-acid glycoprotein concentration in Chinese and Caucasians. *Br J Clin Pharmacol*. 1991;31(5):551-2. doi:10.1111/j.1365-2125.1991.tb05579.x

21. Zhou HH, Adedoyin A, Wilkinson GR. Differences in plasma binding of drugs between Caucasians and Chinese subjects. *Clin Pharmacol Ther*. 1990;48(1):10-7. doi:10.1038/clpt.1990.111

22. Adiwidjaja J, Gross AS, Boddy AV, McLachlan AJ. Physiologically-based pharmacokinetic model predictions of inter-ethnic differences in imatinib pharmacokinetics and dosing regimens. *Br J Clin Pharmacol*. Accessed October 5, 2021. https://bpspubs.onlinelibrary.wiley.com/doi/abs/10.1111/bcp.15084

23. Petain A, Kattygnarath D, Azard J, et al. Population pharmacokinetics and pharmacogenetics of imatinib in children and adults. *Clin Cancer Res*. 2008;14(21):7102-9. doi:10.1158/1078-0432.ccr-08-0950

24. Renard D, Bouillon T, Zhou P, Flesch G, Quinn D. Pharmacokinetic interactions among imatinib, bosentan and sildenafil, and their clinical implications in severe pulmonary arterial hypertension. *Br J Clin Pharmacol*. 2015;80(1):75-85. doi:10.1111/bcp.12584

25. Eechoute K, Fransson MN, Reyners AK, et al. A long-term prospective population pharmacokinetic study on imatinib plasma concentrations in GIST patients. *Clin Cancer Res*. 2012;18(20):5780-7. doi:10.1158/1078-0432.ccr-12-0490
